# Close inbreeding and low genetic diversity in Inner Asian human populations despite geographical exogamy

**DOI:** 10.1038/s41598-018-27047-3

**Published:** 2018-06-20

**Authors:** Nina Marchi, Philippe Mennecier, Myriam Georges, Sophie Lafosse, Tatyana Hegay, Choduraa Dorzhu, Boris Chichlo, Laure Ségurel, Evelyne Heyer

**Affiliations:** 1 Eco-anthropologie et Ethnobiologie, UMR 7206 CNRS, MNHN, Univ Paris Diderot, Sorbonne Paris Cité, Sorbonne Universités, 75016 Paris, France; 2 Republican Scientific Center of Immunology, Ministry of Public Health, Tashkent, 100060 Uzbekistan; 3 Department of biology and ecology, Tuvan State University, Kyzyl, 667000 Russia; 4 Present Address: LM2E-UMR6197, Laboratoire de Microbiologie des Environnements Extrêmes, Institut Universitaire Européen de la Mer, Technopôle Brest-Iroise, Plouzane, 29280 France

## Abstract

When closely related individuals mate, they produce inbred offspring, which often have lower fitness than outbred ones. Geographical exogamy, by favouring matings between distant individuals, is thought to be an inbreeding avoidance mechanism; however, no data has clearly tested this prediction. Here, we took advantage of the diversity of matrimonial systems in humans to explore the impact of geographical exogamy on genetic diversity and inbreeding. We collected ethno-demographic data for 1,344 individuals in 16 populations from two Inner Asian cultural groups with contrasting dispersal behaviours (Turko-Mongols and Indo-Iranians) and genotyped genome-wide single nucleotide polymorphisms in 503 individuals. We estimated the population exogamy rate and confirmed the expected dispersal differences: Turko-Mongols are geographically more exogamous than Indo-Iranians. Unexpectedly, across populations, exogamy patterns correlated neither with the proportion of inbred individuals nor with their genetic diversity. Even more surprisingly, among Turko-Mongols, descendants from exogamous couples were significantly more inbred than descendants from endogamous couples, except for large distances (>40 km). Overall, 37% of the descendants from exogamous couples were closely inbred. This suggests that in Inner Asia, geographical exogamy is neither efficient in increasing genetic diversity nor in avoiding inbreeding, which might be due to kinship endogamy despite the occurrence of dispersal.

## Introduction

At least 10% of humans descend from parents that are related as second cousins or closer, based on registers as well as civil and medical surveys^1^. Such consanguineous mating events can occur for different reasons: it can be because of socio-cultural habits, as emblematically described for European royal families^2^ (mating choice inbreeding), or by chance, for example if there are too many related individuals in the population, as found in isolated groups^3,4^ (drift inbreeding). At the individual scale, the offspring of such close relatives are prone to carry a genetic burden^5^. Indeed, their genomes contain multiple chromosomal segments that are identical by descent^6^ (*i*.*e*., that are inherited from a common ancestor). These segments, called runs of homozygosity (ROHs), consist of a succession of homozygous sites^7^ that subsequently increase the risk for deleterious recessive mutations to be phenotypically expressed^8^ and reduce the fitness at loci under heterozygote advantage^9^. This reduction in fitness associated with inbreeding can result in reduced fertility^10,11^, viability^12,13^ and a wide range of other general disorders^14^. At the population level, inbreeding decreases the effective population size and thus the population genetic variability^15^. A lack of genetic diversity is often associated with a decrease in the adaptive response of a population^16^ and can even lead to its extinction^17,18^. Moreover, in small populations undergoing strong genetic drift, inbreeding can increase the frequency (or even lead to the fixation) of mildly deleterious mutations^19,20^. However, inbreeding can also enable the purge of severely deleterious recessive mutations^21,22^ and eventually improve the population fitness^23^.

How do species cope with such inbreeding issues? Several pre- and post-copulatory behaviours are thought to reduce inbreeding in the animal kingdom^11,24^. Most pre-copulatory behaviours involve kin recognition, which evolved both for avoidance and for cooperation^25,26^. These can rely on the learning of familiarity, which is called Westermarck’s effect in humans^27,28^, on physical and acoustic cues^29–32^ (e.g., human facial kin recognition^33^), or on the expression of some genes such as the human MHC^34^ or mouse MUP^35^. Additionally, in some species, there is a delayed sexual maturation in the presence of relatives from the opposite sex^11,36^. Finally, a more straightforward mechanism, even though it is costly^37^, is to avoid relatives by geographically dispersing^11,38,39^. However, it is unclear if this dispersal is aimed originally at avoiding inbreeding or rather at reducing intra-sexual competition^40^. In western gorillas, females appear to specifically avoid related males when dispersing^41^, which supports the former hypothesis. In humans, geographical exogamy, *i*.*e*., choosing spouses among geographically distant individuals, has been interpreted as a mechanism to avoid marriages between related individuals^42^. One line of evidence supporting this hypothesis comes from the negative relationship observed between exogamy and individual inbreeding in inhabitants from the Orkney Islands of Scotland^43^. Despite this observation, there is a lack of data, whether in humans or in other species, to better understand the general impact of geographical distance between mates on both mating choice inbreeding and drift inbreeding patterns, as well as on population genetic diversity. This is likely because dispersal data are hard to obtain for most species, and combining such data with genetic information is even more challenging^44^.

To explore the impact of geographical exogamy on genetic diversity at both the population and individual scales, we jointly analysed ethnological, geographical and genome-wide data in 16 populations corresponding to 11 distinct ethnic groups from Inner Asia, *i*.*e*., in Uzbekistan, Kyrgyzstan, Tajikistan, West Mongolia and South Siberia. Inner Asia, the region located between the Caspian Sea in the West and Lake Baikal in the East, is particularly interesting in this context, as two groups presenting contrasting cultural traits (notably their language and their matrimonial system) cohabit. The first group, composed of Indo-Iranian-speaking populations, practices mainly geographically endogamous marriages, while the second group, Turkic- and Mongolic-speaking populations (referred to later as Turko-Mongol populations), practices mostly geographically exogamous marriages^45^. Our genomic data allowed us to untangle drift inbreeding, which is due to small population sizes, from mating choice inbreeding, which is due to matrimonial preferences. To our knowledge, this is the first time that such quantitative data have been combined in humans or in any other species.

## Results

### Dispersal behaviours in Inner Asia

**Figure 1:**
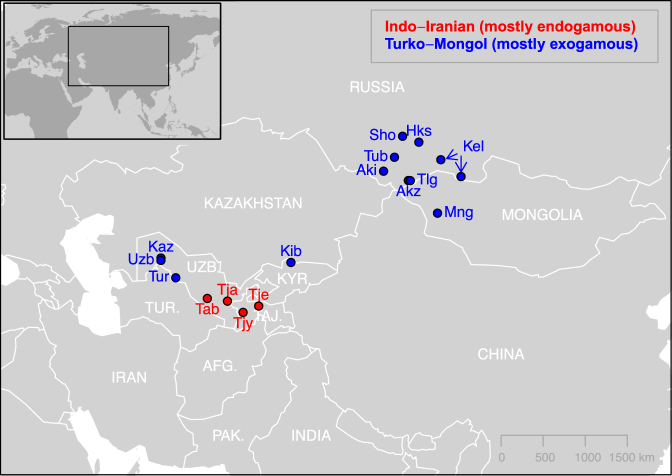
Geographical locations of the 16 Inner Asian populations sampled for this study. On this map generated from *maps*^80^ and *mapdata*^81^ R^82^ packages, the populations are coloured based on their linguistic affiliation, which also correlates with their matrimonial system. Note that the Kel population is composed of Northern Asian Kyrgyz from two different locations, and that Kaz and Uzb, as well as Akz and Tlg, were sampled at the same location, respectively. UZB.: Uzbekistan, KYR.: Kyrgyzstan, TUR.: Turkmenistan, TAJ.: Tajikistan, AFG.: Afghanistan, PAK.: Pakistan. See Supplementary Table 1B for population codes.

**Figure 2:**
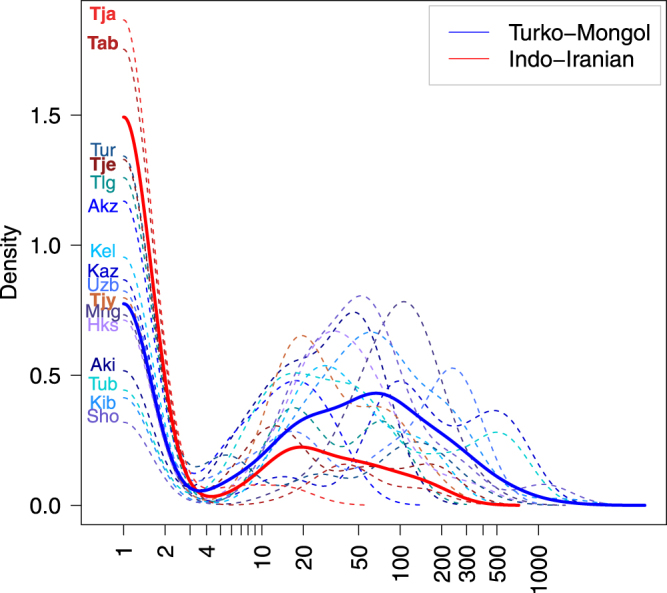
Geographical distances between the birth places of couples in Turko-Mongols and Indo-Iranians. The geographical distances are plotted in log scale (km). Their densities are represented by population (dashed lines) or for the Indo-Iranian and Turko-Mongol groups (solid lines). We represented the average distances within couples per population using a Kernel’s density estimate implemented in R with a smoothing bandwidth of 0.2. See Supplementary Table 1B for population codes.

To quantify the amount of dispersal among spouses, we collected ethno-demographic questionnaires including spatial information for 1,344 individuals (a total of 643 couples) in 16 populations belonging to 11 distinct ethnic groups from Inner Asia and corresponding to 4 Indo-Iranians and 12 Turko-Mongols populations (see Fig. 1 and Supplementary Tables 1A,B). We calculated the distance between the birth places of spouses for each of the 643 couples (Fig. 2); this distance ranged between 0 km for spouses born in the same village (strictly endogamous couples) to 1,474 km, with a median of 5.6 km. We found that Turko-Mongols choose spouses born at larger distances than Indo-Iranians (a median of 17 km and 0 km, respectively; one-tailed Mann-Whitney U, or MWU, test *p-value* < 0.001). Based on the local minimum of the distance densities, we set the limit for geographical exogamy at 4 km and found the percentage of exogamous couples per population to be significantly higher in the 12 Turko-Mongol populations (60% on average, from 33% to 84%) than in the four Indo-Iranians (28% on average, from 6% to 60%) (Supplementary Fig. 1A, Supplementary Table 1, one-tailed MWU test *p-value* = 0.021). When defining exogamy with other thresholds, we still found significant differences between groups at 10, 20 and 50 km (*p-value* < 0.034) and a similar trend for 30 and 40 km (*p-value* = 0.051 and 0.057, respectively). As this result could only be due to differences in average exogamous proportions between groups, we also focused exclusively on exogamous couples (>4 km) and found a tendency for larger distances for Turko-Mongol couples than for Indo-Iranians (60 km and 42 km, respectively, one-tailed MWU test *p-value* = 0.023). Therefore, not only are Turko-Mongol couples geographically more exogamous than Indo-Iranians but they are also composed of spouses born at slightly larger distances.

In addition to the distance within the sampled couples, for each of the 1,344 sampled individuals, we calculated the distance between the birth places of their parents (referred to as the “parental couples”) (Supplementary Fig. 2). As found for the current generation, Turko-Mongols parental couples have higher exogamy rates than those of Indo-Iranians (34% and 11%, respectively; one-tailed MWU test *p-value* = 0.001) (Supplementary Fig. 1B), and these parental distances are significantly larger for Turko-Mongols (one-tailed MWU test *p-value* < 0.001). Furthermore, the exogamy rates of both generations are significantly correlated (Spearman’s *rho* = 0.81; *p-value* = 0.001), even though we observed lower exogamy rates for the parental couples.

### Genetic diversity in Turko-Mongol and Indo-Iranian populations

**Figure 3:**
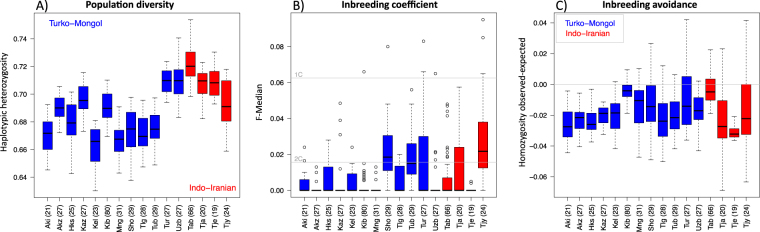
Genetic diversity (**A**) and inbreeding patterns (**B,C**) within populations. Grey lines in (**B**) represent inbreeding values corresponding to second-cousins and first-cousins. The grey line in (**C**) represents the homozygosity population baseline expected under panmixia. The number of samples per population is indicated between parentheses. See Supplementary Table 1B for population codes.

To explore the differences in genetic diversity between Turko-Mongols and Indo-Iranians, we genotyped a subset of 503 individuals among the 16 populations under study (which corresponds to a median of 27 per population) and computed allele-sharing dissimilarity (ASD) distances^46^, a measure based on the proportion of shared alleles between all pairs of individuals within each population (Supplementary Fig. 3). We found a higher median ASD distance for Indo-Iranians than for Turko-Mongols (on average, 0.280 and 0.269, respectively; MWU test *p-value* = 0.029). Because genotyping arrays are known to be enriched for Europeans SNPs^47^ and Indo-Iranians are genetically closer to Europeans than Turko-Mongols^48,49^, the lower diversity observed within Turko-Mongols could be an artefact. To correct for this ascertainment bias, we used another measure of population diversity which is less sensitive to ascertainment bias than site-by-site measures^50,51^: the averaged haplotypic heterozygosity computed for each autosome over low-recombination rate blocks. For this measure, we similarly found significantly higher mean heterozygosities for Indo-Iranians than for Turko-Mongols (on average across autosomes, 0.71 *versus* 0.69, respectively; one-tailed MWU test *p-value* = 0.029) (Fig. 3A, Supplementary Table 1A). Therefore, Turko-Mongol populations overall have a lower genetic diversity than Indo-Iranians, in accordance with their previously reported smaller effective population size^49,52^. These two cultural groups indeed represent two genetic groups with contrasting demographic histories^48,49^, as confirmed in this dataset (see Supplementary Fig. 4, Supplementary Table 2).

### Inbreeding differences between populations

To investigate inbreeding differences between populations, we used a site-by-site inbreeding coefficient (F-Median)^53^ (Fig. 3B) included in the FSuite pipeline^54^ and corresponding to the probability of identity by descent at each marker of the genome^53^. Across populations, 39% of the individuals had positive inbreeding coefficients (from 10 to 90% of each population), with a similar average among Turko-Mongols and Indo-Iranians (39% and 38%, respectively, MWU test *p-value* = 0.86). Moreover, for each of the 503 genotyped individuals, we inferred, from a likelihood ratio test performed by FSuite, the most likely parental mating type, *i*.*e*., inbred mating between first or second cousins, or avunculars, or outbred mating (between less related individuals). Using this likelihood test, we found that 36% of the individuals are inbred across populations with a similar average between Turko-Mongol and Indo-Iranian populations (35% and 36%, respectively; MWU test *p-value* = 0.86). The majority of inbred individuals have parents that are second-cousins: they represent between 5% and 76% of all individuals, depending on the population (Supplementary Table 1A), while individuals with parents being first-cousins were detected only in nine out of the 16 studied populations and represent up to 21% of individuals, without any significant differences between the two cultural groups (MWU test *p-value* = 0.86 and 0.41 for second- and first-cousins, respectively). None of the parents have avuncular relationships, but one case of double-first-cousins was inferred. Overall, the Turko-Mongol and Indo-Iranian groups have a similar distribution of these mating type categories: on average, 31% and 27% of second-cousins, 4% and 7% of first-cousins, and 0% *versus* less than 1% of double-first-cousins (non-significant chi^2^ test *p-value* with Yates’s correction = 0.45), respectively.

We also used runs of homozygosity (ROHs), *i*.*e*., regions of the genome where the paternal and maternal copies are identical and which are long enough to be considered as identical by descent. Their sizes and numbers are therefore informative for the nature of inbreeding^55^. We aimed at distinguishing ROHs of an intermediate size, probably resulting from matings between individuals sharing distant ancestry (and therefore mostly due to drift), from long ROHs likely derived from matings between close relatives (and thus due to matrimonial preferences^43^), even though they could rise from other mechanisms^56,57^. First, using classical thresholds from the literature^43,57–59^, we found that Turko-Mongol individuals have statistically more intermediate ROHs (500–1,500 kb) than Indo-Iranians (52.2 *versus* 36.6; MWU test *p-value* < 0.001; Supplementary Fig. 5A), and these ROHs represent a larger portion of their genomes (44,097 kb *versus* 30,613 kb, respectively; MWU test *p-value* < 10^−36^; Supplementary Fig. 5B). Then, for long ROHs (>1,500 kb), we observed that the Turko-Mongol individuals have more ROHs than Indo-Iranian individuals (10.9 *versus* 9.0, respectively; MWU test *p-value* = 10^−6^) (Supplementary Fig. 5C), suggesting more matings between closely related individuals in Turko-Mongols. This is not in agreement with our results from FSuite, where we did not detect any significant difference in mating choice inbreeding between groups. However, when we instead defined boundaries per population with a model-based clustering algorithm as conducted in Pemberton *et al*.^51^, we obtained different thresholds for the ROH classes: on average, class B (intermediate) ROHs range between 885 and 2,647 kb, while class C (long) ROHs are above 2,647 kb (Supplementary Table 1A). Using these new boundaries, we did not detect differences either for the number or for the total length of class C-ROHs between Turko-Mongols and Indo-Iranians (MWU test *p-value* = 0.14 and 0.24, respectively, Supplementary Fig. 5C,D), but we still found more and longer class B-ROHs in Turko-Mongols (28.3 *versus* 20.1, respectively; MWU test *p-value* < 10^−22^; and 36,340 *versus* 26,389 kb, respectively; MWU test *p-value* = 10^−17^, Supplementary Fig. 5A,B).

### Inbreeding avoidance by geographical dispersal?

To investigate inbreeding avoidance, we compared the observed homozygous proportion of each individual genome to the expectation under random mating^60^. In each population, we found most individuals to be less homozygous than expected: overall, the median differences between observed and expected homozygosity are negative and are between −0.032 and −0.004 (average of −0.019), without any significant difference between Indo-Iranians and Turko-Mongols (MWU test *p-value* = 0.31; Fig. 3C). To test whether this result is restricted to Inner Asia, we analysed 11 worldwide populations from HapMap3 and found that they also have negative median values, except for the Gujarati Indians from Houston (GIH), who have a median of 1.6 * 10^−6^ (Supplementary Fig. 6).

We then tested whether exogamous populations include less inbred individuals than endogamous ones as expected if geographical dispersal is a mechanism of inbreeding avoidance. Surprisingly, in our dataset, the exogamy rate per population (defined with a limit of 4 to 50 km) is significantly correlated neither with the percentage of closely inbred individuals nor with the proportion of first-cousins’ descendants (Spearman’s correlation test *p-value* > 0.1 for all populations, only Turko-Mongols or only Indo-Iranians). Similarly, in terms of genetic diversity, the population exogamy rates are not associated with haplotypic heterozygosities (Spearman’s correlation test: *p-value* > 0.29 for Turko-Mongols and *p-value* = 0.75 for Indo-Iranians). We further verified these results when taking into account potential confounding factors such as the age and sex ratio within each population, various environmental variables (altitude, ecosystem), as well as lifestyle (urban/rural and sedentary/nomadic) (Supplementary Table 1B). Considering the effect of each variable one at a time, we found that only the ecosystem had a significant effect on the percentage of inbred individuals per population while both the sedentary/nomadic, ecosystem and sex-ratio variables had a significant effect on the haplotypic heterozygosity (linear regressions, *p-values* < 0.05). However, when using multiple linear regression models and ANOVAs taking into account various combinations of these significant variables, we never found the exogamy rate to be significantly correlated with the percentage of inbred individuals per population nor with the haplotypic heterozygosity (*p-values* > 0.10).

**Figure 4:**
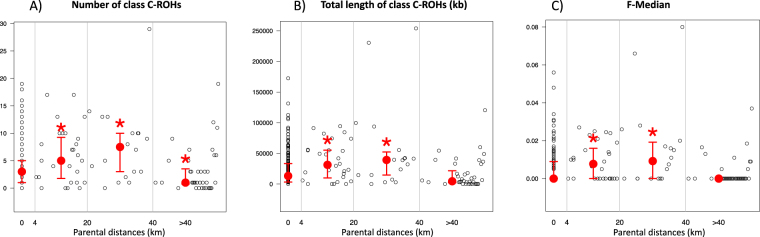
Parental couple distances and close inbreeding patterns for descendants from Turko-Mongols. For each of three estimators of inbreeding, we show in red the median value for four different classes of parental distances (<4 km, 4–20 km, 20–40 km and <40 km) as well as confidence intervals at 25% and 75%. The results of a MWU test comparing the <4 km class to all three others is shown below the plot.

We further explored the correlation between exogamy and inbreeding at the individual scale, *i*.*e*., analysing the relationship between the mating choice inbreeding pattern of each individual and the geographical distance between his/her parents’ birth places. This was only done in Turko-Mongols, as there were too few descendants from exogamous couples in Indo-Iranians. Interestingly, as seen in Fig. 4, we observed that the relationship between mating choice inbreeding and geographical distances between parents is not linear. Indeed, we found that descendants from exogamous couples born 4 to 40 km apart have significantly higher inbreeding levels than descendants from endogamous couples (≤4 km) (whether measured as the number or total length of class C-ROHs or as the F-Median: MWU test *p-values* < 0.05). Then, inbreeding decreases such that descendants from long-range exogamous couples (>40 km) are as inbred as descendants from endogamous couples for class C-ROH total length and F-Median (MWU test *p-values* > 0.05) and significantly less inbred for the number of class C-ROHs (MWU test *p-value* = 0.03). Consistently, we found that the distance between exogamous spouses (in log scale) had an effect on their descendant’s inbreeding (for number and total length of class C-ROHs and F-Median coefficient, Spearman’s *rho* between −0.36 and −0.32, *p-values* < 3 * 10^−4^) (Supplementary Fig. 7).

Overall, among descendants from exogamous couples (>4 km), 30% have parents that are second-cousins, and 7% are first-cousins, which are proportions that do not differ from those obtained for endogamous couples (26% and 5%) (Supplementary Fig. 8).

## Discussion

Two cultural groups, which matrimonial systems are reported to differ, coexist in Inner Asia: Turko-Mongols are described as mainly exogamous while Indo-Iranians are thought to be mainly endogamous^45^. However, it is not always clear if exogamy refers to clan (ethnic) or village (geographical) exogamy. Here, we used a dataset of 16 populations representing 11 different ethnic groups from both cultural groups and we quantified geographical exogamy rates and distances in each population. Using an empirical threshold of 4 km, we confirmed that matrimonial behaviours differ as described in the literature, even though we found some exceptions: three Turko-Mongol populations (out of 14) have less than 50% exogamy, whereas one Indo-Iranian population (out of four) has more than 50% exogamy. Of note, this latter Indo-Iranian exception is a peculiar Tajik population (Tjy) that underwent population displacement in the 1970s from the Yagnob Valley to the Dushanbe area (approximately 60 km apart) in Tajikistan^61^. In general, the exogamous rates measured for the parental and current generations are highly correlated, even though we observed a trend towards higher values in the current generation, possibly reflecting changes in matrimonial behaviours through time (e.g., linked to urbanisation) or some recall bias for information about parental couples.

We used these contrasted dispersal behaviours to investigate the level of inbreeding in these populations and to test whether geographical exogamy is a mechanism of inbreeding avoidance. First, we showed that inbreeding resulting from matings between relatives that are at least second-cousins indeed occurs in Inner Asia: on average, 36% of the individuals are inbred. This estimation exceeds the worldwide lower bound estimate of 10.4%^1^ but is still within the range of estimations made for neighbouring regions (28.6% for Turko-Mongol and Tajik populations from Xinjiang; 35.7% in South Asia) from data available in http://consang.net/index.php/Global_prevalence. Interestingly, marriages between first-cousins that are prescribed in some Muslim societies from areas close to Inner Asia, such as Middle East^62^ or Pakistan^63^, only represent a small proportion of the Inner Asian marriages despite their Muslim tradition. Furthermore, most of the mating choice inbred alliances in our dataset are between second-cousins, and no avuncular relationship was observed. As inbreeding information is lacking for Inner Asia, our data complete the global picture of mating choice inbreeding prevalence. Interestingly, among the 16 studied populations, we found a large variability of mating choice inbreeding level (from 5% to 83%) and of mating type proportions. In particular, three populations (Sho, Tub and Tjy) include more than 50% of inbred individuals (79%, 83% and 79%, respectively). Of all the factors we tested (altitude, ecosystem, rural/urban, nomadic/sedentary lifestyle, age or sex-ratio within populations), only the ecosystem had a significant effect on the rate of inbreeding, with the forest environment (*i*.*e*., the Sho and Tub populations) being significantly associated with a higher inbreeding than the other environments. Further data would be needed to explore these relationships. Moreover, the population effective population size, as approximated by haplotypic heterozygosity, was not correlated with the level of close inbreeding observed in the populations, based on the proportions of i) inbred individuals per population, ii) first-cousins’ descendants, and on iii) the population median F-Median (Spearman’s correlation test *p-values* > 0.2).

From a methodological point of view, our study highlights the necessity to define for each population its own ROH boundaries rather than using arbitrary thresholds originally defined for European populations (*i*.*e*. between 500 and 1,500 kb and over 1,500 kb, respectively). Indeed, we detected some inconsistencies between the two approaches: the classes B and C-ROHs ranges only partly overlap with the arbitrary thresholds ranges, and the limit between class B and C is about two times larger than the 1,500 kb threshold. Consequently, the results obtained for ROHs longer than 1,500 kb are contradictory with those obtained for class C-ROHs and with the results obtained based on FSuite.

Another result of our study is that the observed inbreeding levels in all Inner Asian populations deviate from the expectation under random mating, with each population being composed of less inbred offspring than expected by chance. We found the same result in nearly all populations from the HapMap3 worldwide dataset, which suggests that this is a general pattern. Such a deficit of inbreeding could be due to the avoidance of close kin matings (*i*.*e*., brother-sister) that are proscribed in all societies^64^ or to other pre/post-copulatory mechanisms, and this remains an open question.

An additional important result of our study is that geographical distances are not negatively correlated with inbreeding, as could have been expected under an isolation-by-distance model^65^. Interestingly, a recent study based on a large genealogical dataset, collected across Western Europe and North America, and including birth places information, similarly found an absence of correlation between relatedness and the distance between couples, for the cohorts born before 1850^66^. Our analyses within present-day Turko-Mongols reveal more specifically that the structure of the relationship between geographical distance and mating choice inbreeding is not linear, but rather tends to be bell-shaped, and thus cannot be correctly assessed with a single correlation test. Indeed, descendants from parents born 4 to 40 km apart are more inbred than descendants from endogamous couples (≤4 km) or from long-range exogamous ones (>40 km). As a consequence, close inbreeding exists despite geographical exogamy, and about a third of descendants from exogamous couples are inbred.

These results, in addition to those obtained by^66^, highlight the importance of using geographic distances rather than exogamy rates to characterize the impact of exogamy on inbreeding, as already described when studying patrilocality^67^. Indeed, when we compare mating choice inbreeding patterns for descendants from exogamous and endogamous couples defined for thresholds of 4, 10, 20 and 30 km, we find no significant differences (for number and total length of class C-ROHs and F-Median coefficient: MWU test *p-values* > 0.1). We only detect significantly lower values in descendants from exogamous couples for larger distances above 40 and 50 km (*p-values* < 0.03).

Such inbred matings of individuals born at intermediate distances can be explained by two scenarios. First, spouses could be chosen outside the village unit but still within a quite genetically homogeneous unit^68,69^. However, under this hypothesis, we would expect inbreeding patterns to be similar between descendants of endogamous and intermediate exogamous couples, yet we observe a significant excess of mating choice inbreeding in the latter (4 to 40 km apart). A second scenario is that geographical exogamous marriages preferentially involve spouses from the same family (kinship endogamy). It is indeed known from previous ethnological work that in patrilineal societies where individuals cannot mate with related spouses from the paternal line, they often choose spouses among relatives from the maternal side^70^. To properly examine this hypothesis in the particular case of Turko-Mongols, further analyses and sampling are required. Indeed, we would need to analyse genetic data for more exogamous couples as well as for individuals coming from the populations of origin of both spouses. Thereby, we would be able to explore spouses’ kinship by comparison with other potential partners and genetic distances between the populations. Furthermore, based on the kinship estimated from the X chromosome, we could investigate if related partners belong to the same maternal line, as assumed for these patrilineal societies, which would be of great interest to understand matrimonial strategies.

Our results also challenge the intuition that exogamy necessarily increases the genetic diversity within a population and therefore reduces drift inbreeding. Indeed, we found that Turko-Mongol populations have a lower genetic diversity (as measured by the mean haplotypic heterozygosity) and more intermediate ROHs associated with drift inbreeding than those of Indo-Iranians despite higher exogamous rates. However, because these groups have different demographic histories (see Supplementary Fig. 4) and effective population sizes^52^, we chose to focus on each group separately. Within each group, we also found that across populations, the exogamy rates are not correlated with haplotypic heterozygosities. Moreover, at the individual scale, the total length or number of class B-ROHs was not significantly different between descendants from endogamous and exogamous Turko-Mongol couples (MWU test *p-values* = 0.7 and 0.9, respectively). This result is contrary to what was observed in the Orkney Islands of Scotland^43^; thus, this correlation might differ depending on the cultural context.

Overall, this research sheds light on mating choice preferences: we showed that two thirds of partners that have not dispersed did mate with unrelated individuals, and that drift and mating choice inbreeding is variable, even among close-by populations. We also provide new insights into the relationship between dispersal and inbreeding in humans, based on genetic data, and demonstrate that geographical exogamy is not necessarily negatively associated with mating choice inbreeding, but rather can have a more complex non-linear relationship. Contrary to the common situation in many animals, this finding suggests that Inner Asian human populations who practise exogamy at small geographical scales might be focused on alliance strategies that result in kinship endogamy. To test whether these patterns depend on the cultural context, these questions should be addressed in other human populations, for example, Canadian populations^71^ or European isolates^72^ for whom genealogical detailed data and genomic data are already available. In such populations where phenotypic data are also available, inbreeding patterns can further be used to map disease-associated mutations^73,74^. More generally, it would be of great interest to elucidate the variability of mating choice among cultures by systematically investigating inbreeding, which is generally disregarded in human demographic studies^75,76^.

## Materials and Methods

### Population samples

We collected data during several field expeditions conducted in Inner Asia between 2001 and 2012. We defined populations as groups of individuals living in a similar area and belonging to the same ethnic group, based on the self-reported spoken native language. We obtained geographical and ethnological information (birth place and home language) for 1,344 individuals (a total of 643 couples) and their parents in 16 Inner Asian populations, *i*.*e*. 12 Turko-Mongol populations and four Indo-Iranian populations (Fig. 1, Supplementary Table 1B). Among these, we collected DNA from blood or saliva in 907 individuals. Written informed consent was obtained for all participants, and our sampling and analyses followed ethical principles approved by the Museum National d’Histoire Naturelle (France), Ministry of Health (Altai Republic), Tuvan State University (Republic of Tuva), Khovd University (Mongolia) and Uzbek Academy of Sciences (Uzbekistan).

### Geographical exogamy

We inferred geographical exogamy from the geographical distance between the birth places of spouses (see Supplementary Materials and Methods). These distances can be calculated between each husband and wife (*i*.*e*. current generation) or between the parents of the husband on the one hand, and those of the wife on the other hand (*i*.*e*. parental generation). We defined exogamous couples as those with spouses born at more than 4 km (average population local minima based on Fig. 2), and we also tested for other definitions of exogamy with arbitrary thresholds at 10, 20, 30, 40, and 50 km.

### Genetic diversity

Among the 907 individuals with blood or saliva sampled, we extracted DNA and genotyped 576 of them on five different Illumina arrays (either 660W-Quad, OmniExpress, Omni1-Quad, Omni2.5, or Omni5Exome). We chose the individuals randomly to reach an average of approximately 30 individuals per population. Among them, 503 passed quality-control filters (see Supplementary Materials and Methods and Supplementary Table 3) and were unrelated (*i*.*e*., we excluded 36 individuals found to be more related than first-cousins), which resulted in a median of 27 individuals per population (66% of males and 34% of females, Supplementary Table 1). After merging the arrays, we obtained data for 253,532 SNPs, including 105,858 independent SNPs (r^2^ < 0.5). The SNPs data has been deposited at the European Genome-phenome Archive (EGA), under accession number EGAS00001002951. We computed allele-sharing dissimilarity (ASD) distances^77^ between individuals using the software *asd*^46^ (see Supplementary Materials and Methods). We also calculated the autosomal haplotypic heterozygosity over low-recombination rated blocks for each population based on^78^ (see Supplementary Materials and Methods).

### Inbreeding coefficients

For each individual, we estimated the inbreeding coefficient (F-Median) which is the median value of multiple estimates of the probability of identity by descent at each marker of the genome^53^, with FSuite v1.0.3^54^. We estimated it by generating 100 subsets of independent SNPs (>0.5 cM)^55^, and computing the median of these 100 coefficients. Furthermore, a likelihood ratio test was performed to assign each individual as inbred or outbred. For each individual, based on both F-Median and A-Median, it was possible to estimate the probability to be the descendant of avunculars (AV), double first-cousins (2x1C), first-cousins (1C), second-cousins (2C), or unrelated individuals (OUT; defined as less related than second-cousins). We kept the most likely inbred mating type for each individual. We also calculated the genomic excess of homozygosity^60^ relative to an expected baseline of homozygosity for each of the Inner Asian populations and for the 11 populations from the HapMap3 worldwide dataset using plink1.9 (see Supplementary Materials and Methods). Furthermore, we identified runs of homozygosity, called ROHs^3^, using plink1.9 (see Supplementary Materials and Methods). First, to be able to compare with other studies from the literature^43,57–59^, we categorized ROHs based on classical thresholds: 500–1,500 kb for intermediate ROHs, *versus* > 1,500 kb for long ROHs. However, as Pemberton *et al*.^3^ showed that these ROH thresholds can be variable between populations, we also used population-specific categories defined from their ROH size classification method (*i*.*e*. based on three-component Gaussian fitting of the ROH length distribution, using Mclust from the *mclust* package (v.5^79^) in R) resulting in what we call intermediate (class B-ROHs) and long ROHs (class C-ROHs). For each of the four ROH classes, we computed the number of ROHs observed within an individual genome and their total length.

### Data Availability

The datasets generated and analysed during the current study are available from the corresponding author upon reasonable request.

## Electronic supplementary material

Supplementary Information
