# Supplementary material for: Close inbreeding and low genetic diversity in Inner Asian human populations despite geographical exogamy
Source: Sci Rep. 2018 Jun 20;8:9397. doi: 10.1038/s41598-018-27047-3 (PMC6010435; doi:10.1038/s41598-018-27047-3)
Supplement: Supplementary file 1 — Supplementary Information [file 41598_2018_27047_MOESM1_ESM.pdf]

## Supplementary Information

# Close inbreeding and low genetic diversity in Inner Asian human populations despite geographical exogamy

Authors: Nina Marchi[a*], Philippe Mennecier[a], Myriam Georges[a,b], Sophie Lafosse[a], Tatyana Hegay[c], Choduraa Dorzhu[d], Boris Chichlo[a], Laure Ségurel[a,#], Evelyne Heyer[a,#]

*#Co-supervised this work*

## Supplementary Materials and Methods

### Geographical exogamy

To infer the geographical distance between the birth places of spouses (collected by the use of questionnaire available at the end of this document), we obtained the geographical coordinates of each location and calculated the pairwise great-circle distances with an own designed python3 code using the Spherical Law of Cosines formula and an Earth radius of 6,367.445 km. Based on the population local minima on Figure 2 (comprised between 2 km and 12 km, with an average 4 km, while Tja, a population that did not have a local minimum, was excluded), we defined exogamous couples as those with spouses born at more than 4 km. We also tested for other definitions of exogamy with arbitrary thresholds at 10, 20, 30, 40, and 50 km. Note that for three out of 16 populations (Kaz, Tur and Uzb), we had no information for the parental generation.

### SNP genotyping and quality control

All individuals were genotyped on an Illumina genotyping array (either 660W-Quad, OmniExpress, Omni1-Quad, Omni2.5, or Omni5Exome; see details in Supplementary Table 3). Genotyping was performed by the "Plate-forme Post-Génomique de la Pitié-Salpétrière (P3S)" or by the "Institut Pasteur – Genopole (Génotypage des Eucaryotes)", in Paris, France. For two of the arrays (Illumina Omni1-Quad and Omni2.5), we performed our own genotype-calling quality control whereas for the others, the quality control was done by the sequencing platform. Indeed, for each array separately, we performed a three-stage quality control procedure inspired from [1], restricting our analyses to autosomal SNPs (*i.e.,* excluding CNVs and non-autosomal markers). Briefly, we excluded SNPs with ambiguous genomic position, SNPs that failed genotyping in the sample set, SNPs with a call rate below 90% or a cluster separation below 0.2, SNPs without ID in rs nomenclature, SNPs found to be duplicates, indels and monomorphic SNPs. We removed samples with more than 15% of missing data. Then, we performed a population-genetic quality control following [1]. We first identified pairs of samples more related than first cousins using

Relpair 2.0.1 software [2,3]. We performed this analysis on three non-overlapping subsets of SNPs in Hardy-Weinberg equilibrium (*p-value*>$10^{-5}$ for each population and >$10^{-2}$ for each pair of populations) and bimorphic in every population. For pairs of individuals closer than first cousins, we removed the individual with the lowest genotyping-call or the individual involved in the highest number of relations, resulting in 52 individuals being excluded. Finally, we removed the monomorphic SNPs within each array. At the end, for the five arrays, we obtained data for 526,823 to 2,609,107 SNPs genotyped for the 519 samples of interest.

To generate a joint dataset, we manually merged the five datasets based on SNPs rs. To control that alleles are coded on the same strand between arrays, we removed A-T and C-G polymorphisms, and SNPs that were triallelic on the two arrays, resulting in a dataset of 253,606 SNPs. We found three pairs of relatives within the merged dataset, and as previously described, we excluded three samples, in addition to 13 samples with a call-rate < 95%, leading to a total of 503 individuals. 292 of these individuals genotyped on the Omni1 and Omni2.5 arrays have already been published in [4], while the 211 other individuals were newly published in our present study. Finally, we excluded SNPs in Hardy-Weinberg disequilibrium (plink1.9 [5] function *--hardy*, *p-value*>$10^{-5}$), leading to a final dataset of 253,532 SNPs. Within this dataset, we generated a subset of 105,858 independent SNPs with $r^2<0.5$ (plink1.9 function *--indep* 50 5 2).

**Genetic diversity**

We computed the matrix of allele-sharing dissimilarity between all individuals for the independent SNPs dataset, using the software *asd* [6]. We also computed pairwise $F_{ST}$ distances for each pair of populations on the independent SNPs dataset using ArlSumStat [7] (Supplementary Table 2). We present only $F_{ST}$ distances on the independent SNPs as they are highly correlated to those based on the whole SNP dataset (Spearman's *rho*=0.997, *p-value*<$2.2*10^{-16}$). We represented both the ASD and $F_{ST}$ pairwise distance matrices as the two first dimensions of

MultiDimensional Scaling (MDS) performed with R function *cmdscale* [8]. To evaluate whether the first two MDS dimensions represented accurately the whole matrix, we calculated the Spearman's rank-sum correlation *rho* between the MDS distances on dimensions 1 and 2, and their corresponding $F_{ST}$ and ASD distances, respectively.

We calculated the haplotypic heterozygosity of each population based on [1]. We first reconstructed non-overlapping blocks of SNPs in high linkage disequilibrium (LD) using the whole SNPs dataset. We defined blocks of LD as intervals of 5-15 SNPs where the recombination rate is below 0.5cM/Mb between each pair of contiguous SNPs (using the GRCh37 recombination map). We generated 2,267 such blocks of LD, with on average 103 blocks per chromosome (from 24 on chromosome 21 to 222 on chromosome 1). These blocks include 13,340 SNPs, *i.e.,* 5.3% of the total dataset.

Then we phased these blocks with plink1.7 [9] *--hap* function, that uses an expectation-maximization phasing algorithm. For each population, we estimated the frequencies of the haplotypes (plink1.7 *--hap-freq* function), calculated the heterozygosity of each haplotype based on Nei's formula and computed the mean haplotypic heterozygosity along each chromosome:

$$H_{k\text{-chromosome}}=\frac{\frac{2Npop}{2Npop-1}*\sum_{j=1}^{Nblocks}(1-\sum_{i=1}^{Nalleles}f_{i,j}{}^{2})}{Nblocks}.$$

**Inbreeding coefficients and parental mating types**

We chose to use FSuite to infer the inbreeding coefficient, as it is efficient to infer distant relationships (1C and 2C type) [10], even in small-sized samples [11]. We used the software's default option that creates 100 random submaps with one marker every 0.5 cM [11].

We calculated the proportion of homozygous sites within each individual genome, relative to an expected baseline of homozygosity per population, using plink1.9 *--het* function on the independent SNPs dataset [9] without any options. Thus our results were obtained without the Nei

and Roychoudhury's correction, implemented the *--small-sample* option. For comparison, we also computed it for the 11 populations from the HapMap3 worldwide dataset. First, we removed 236 first-degree relatives, and SNPs that were non-autosomal, monomorphic or deviating from Hardy-Weinberg equilibrium (*p-value*$<10^{-5}$ in at least one population, and $<10^{-2}$ in at least two populations) [12], to eventually generate a dataset of 195,874 independent SNPs ($r^2<0.5$, plink1.9 function *--indep 50 5 2*).

We identified runs of homozygosity, called ROHs [13], within each individual genome based on the whole SNPs dataset, using the plink1.9 *--homozyg* function with by-default options but changing the options *--homozyg-kb 20 --homozyg-snp 50* as done in [14] to be able to detect ROHs contained more than 50 SNPs (rather than 100 by default) for a length larger than 20 kb.

**Pipeline for quality control and datasets merging.**

The pipeline includes three stages of analysis. Briefly, the first step cleans SNPs and samples with a low quality or in duplicate; the second step estimates the genetic relatedness between samples and the third steps remove relatives closer than 1st cousins as well as monomorphic SNPs. Then the five arrays were merged, quality and relatedness were controlled one more time and other individuals included in these arrays but not from Inner Asia were removed. Moreover, we added a Hardy-Weinberg equilibrium test on the SNPs dataset. An additional step generates a subset of independent SNPs.

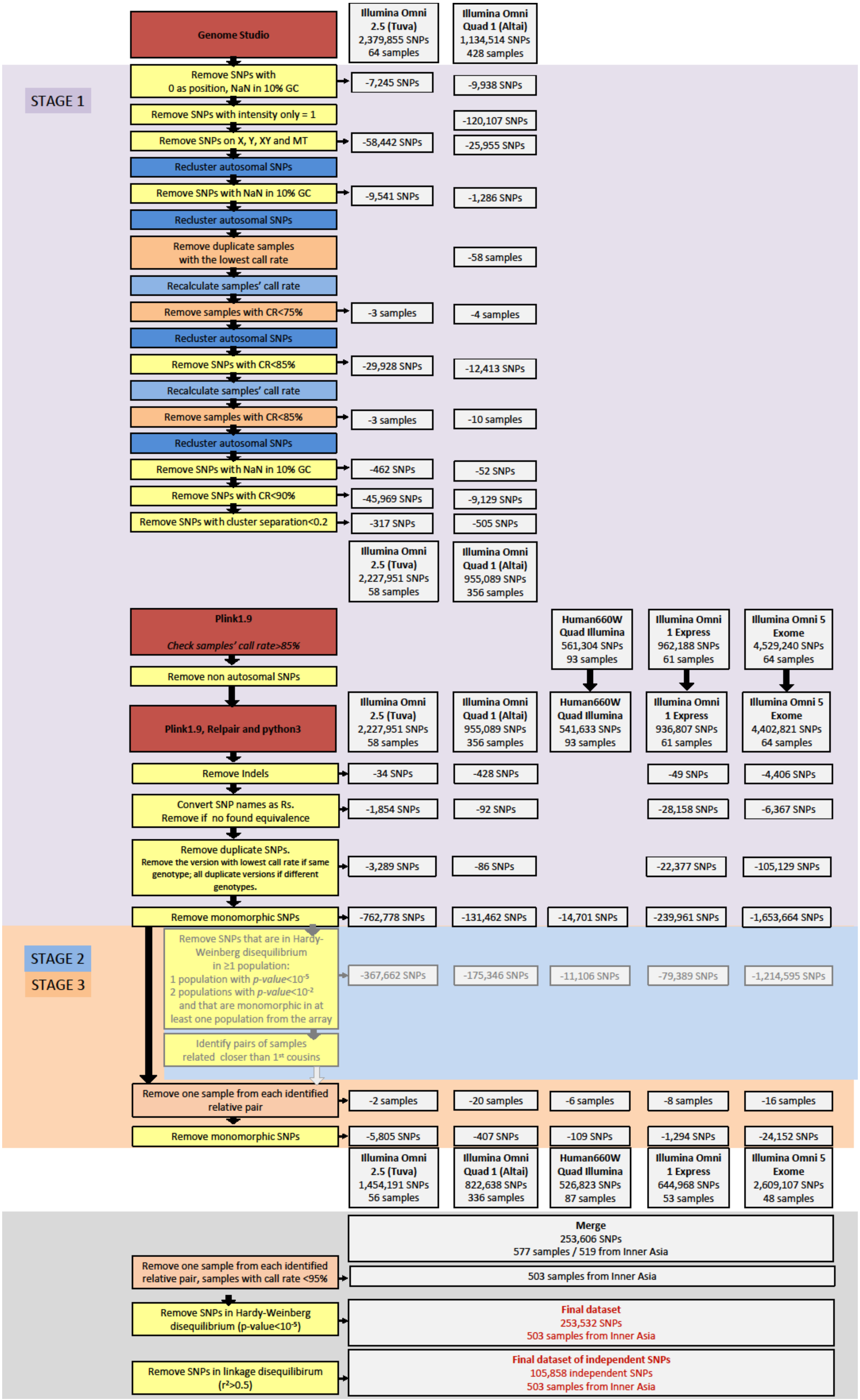

Genome Studio
Illumina Omni 2.5 (Tuva) 2,379,855 SNPs 64 samples
Illumina Omni Quad 1 (Altai) 1,134,514 SNPs 428 samples
STAGE 1
Remove SNPs with 0 as position, NaN in 10% GC
-7,245 SNPs
-9,938 SNPs
Remove SNPs with intensity only = 1
-120,107 SNPs
Remove SNPs on X, Y, XY and MT
-58,442 SNPs
-25,955 SNPs
Recluster autosomal SNPs
Remove SNPs with NaN in 10% GC
-9,541 SNPs
-1,286 SNPs
Recluster autosomal SNPs
Remove duplicate samples with the lowest call rate
-58 samples
Recalculate samples' call rate
Remove samples with CR<75%
-3 samples
-4 samples
Recluster autosomal SNPs
Remove SNPs with CR<85%
-29,928 SNPs
-12,413 SNPs
Recalculate samples' call rate
Remove samples with CR<85%
-3 samples
-10 samples
Recluster autosomal SNPs
Remove SNPs with NaN in 10% GC
-462 SNPs
-52 SNPs
Remove SNPs with CR<90%
-45,969 SNPs
-9,129 SNPs
Remove SNPs with cluster separation<0.2
-317 SNPs
-505 SNPs
Illumina Omni 2.5 (Tuva) 2,227,951 SNPs 58 samples
Illumina Omni Quad 1 (Altai) 955,089 SNPs 356 samples
Plink1.9
Check samples' call rate>85%
Human660W Quad Illumina 561,304 SNPs 93 samples
Illumina Omni 1 Express 962,188 SNPs 61 samples
Illumina Omni 5 Exome 4,529,240 SNPs 64 samples
Remove non autosomal SNPs
Plink1.9, Relpair and python3
Illumina Omni 2.5 (Tuva) 2,227,951 SNPs 58 samples
Illumina Omni Quad 1 (Altai) 955,089 SNPs 356 samples
Human660W Quad Illumina 541,633 SNPs 93 samples
Illumina Omni 1 Express 936,807 SNPs 61 samples
Illumina Omni 5 Exome 4,402,821 SNPs 64 samples
Remove Indels
-34 SNPs
-428 SNPs
-49 SNPs
-4,406 SNPs
Convert SNP names as Rs. Remove if no found equivalence
-1,854 SNPs
-92 SNPs
-28,158 SNPs
-6,367 SNPs
Remove duplicate SNPs. Remove the version with lowest call rate if same genotype; all duplicate versions if different genotypes.
-3,289 SNPs
-86 SNPs
-22,377 SNPs
-105,129 SNPs
Remove monomorphic SNPs
-762,778 SNPs
-131,462 SNPs
-14,701 SNPs
-239,961 SNPs
-1,653,664 SNPs
STAGE 2
STAGE 3
Remove SNPs that are in Hardy-Weinberg disequilibrium in ≥1 population: 1 population with p-value<10⁻⁵ 2 populations with p-value<10⁻² and that are monomorphic in at least one population from the array
-367,662 SNPs
-175,346 SNPs
-11,106 SNPs
-79,389 SNPs
-1,214,595 SNPs
Identify pairs of samples related closer than 1st cousins
Remove one sample from each identified relative pair
-2 samples
-20 samples
-6 samples
-8 samples
-16 samples
Remove monomorphic SNPs
-5,805 SNPs
-407 SNPs
-109 SNPs
-1,294 SNPs
-24,152 SNPs
Illumina Omni 2.5 (Tuva) 1,454,191 SNPs 56 samples
Illumina Omni Quad 1 (Altai) 822,638 SNPs 336 samples
Human660W Quad Illumina 526,823 SNPs 87 samples
Illumina Omni 1 Express 644,968 SNPs 53 samples
Illumina Omni 5 Exome 2,609,107 SNPs 48 samples
Merge 253,606 SNPs 577 samples / 519 from Inner Asia
Remove one sample from each identified relative pair, samples with call rate <95%
503 samples from Inner Asia
Remove SNPs in Hardy-Weinberg disequilibrium (p-value<10⁻⁵)
Final dataset 253,532 SNPs 503 samples from Inner Asia
Remove SNPs in linkage disequilibirum (r²>0.5)
Final dataset of independent SNPs 105,858 independent SNPs 503 samples from Inner Asia

№ .......... №  ...........

**Inspection site** ......................................................... **Date** ........../08/2012

| **M** | **F** |
|---|---|
| **Name**................................................................................<br>**Date Of Birth…** Place of Birth<br>Child order in the family .................<br>Son order ......................................... | **Name**................................................................................<br>**DOB …………….** Place of Birth<br>Child order in the family .................<br>Girl order ......................................... |

**Your spouse**

| | | |
|---|---|---|
| Alive: ...........Yes / No | First marriage : ..... Yes / No | (Который : .... ) |
| Are you related to your spouse ? .............................................................. Yes / No | | |

**M** **Location** **F**

| | |
|---|---|
| Place of Birth ......................................................................... | Place of Birth ......................................................................... |
| Domicile ............................................. , when ? .................... | Domicile ............................................. , when ? .................... |
| Domicile of father .................................................................<br>Paternal grandfather .........................................<br>Paternal grandmother ....................................... | Domicile of father .................................................................<br>Paternal grandfather .........................................<br>Paternal grandmother ....................................... |
| Domicile of mother.................................................................<br>Maternal grandfather ........................................<br>Maternal grandmother ...................................... | Domicile of mother.................................................................<br>Maternal grandfather ........................................<br>Maternal grandmother ...................................... |

**M** **Language** **F**

| | |
|---|---|
| Your language ........................................................................ | Your language ........................................................................ |
| Father’s language ...................................................................<br>Paternal grandfather .........................................<br>Paternal grandmother ....................................... | Father’s language ...................................................................<br>Paternal grandfather .........................................<br>Paternal grandmother ....................................... |
| Mother’s language .................................................................<br>Maternal grandfather ........................................<br>Maternal grandmother ...................................... | Mother’s language .................................................................<br>Maternal grandfather ........................................<br>Maternal grandmother ...................................... |

**M** **Tribe** **F**

| | |
|---|---|
| Your tribe ............................................................................... | Your tribe ............................................................................... |
| Father’s tribe .........................................................................<br>Paternal grandfather .........................................<br>Paternal grandmother ....................................... | Father’s tribe .........................................................................<br>Paternal grandfather .........................................<br>Paternal grandmother ....................................... |
| Mother’s tribe ........................................................................<br>Maternal grandfather ........................................<br>Maternal grandmother ...................................... | Mother’s tribe ........................................................................<br>Maternal grandfather ........................................<br>Maternal grandmother ...................................... |

**.../...**

**Married sons and daughters**

| | | |
|---|---|---|
| Total amount of children (including died) ............................................ | Amount of daughters (including died) ........................................................... | Amount of sons (including died) ...... |
| Total amount of married sons and daughters ...................................... | Amount of married daughters ......... | Amount of married sons .................... |
| Is last child married ? Yes / No | | Age of last child .............................. |

| | | | |
|---|---|---|---|
| **Eldest child :** | Sex: M / F | DOB ........................ | Domicile ............................................................. |
| Spouse : | Date of birth ............................................. | Language .......... | Tribe ........................... |
| Are the spouse related ? Yes / No | | | |

| | | | |
|---|---|---|---|
| **Second child :** | Sex: M / F | DOB ........................ | Domicile ............................................................. |
| Spouse : | Date of birth ............................................. | Language .......... | Tribe ........................... |
| Are the spouse related ? Yes / No | | | |

| | | | |
|---|---|---|---|
| **Third child :** | Sex: M / F | DOB ........................ | Domicile ............................................................. |
| Spouse : | Date of birth ............................................. | Language .......... | Tribe ........................... |
| Are the spouse related ? Yes / No | | | |

| | | | |
|---|---|---|---|
| **Fourth child :** | Sex: M / F | DOB ........................ | Domicile ............................................................. |
| Spouse : | Date of birth ............................................. | Language .......... | Tribe ........................... |
| Are the spouse related ? Yes / No | | | |

| | | | |
|---|---|---|---|
| **Fifth child :** | Sex: M / F | DOB ........................ | Domicile ............................................................. |
| Spouse : | Date of birth ............................................. | Language .......... | Tribe ........................... |
| Are the spouse related ? Yes / No | | | |

| | | | |
|---|---|---|---|
| **Sixth child :** | Sex: M / F | DOB ........................ | Domicile ............................................................. |
| Spouse : | Date of birth ............................................. | Language .......... | Tribe ........................... |
| Are the spouse related ? Yes / No | | | |

| | | | |
|---|---|---|---|
| **Seventh child :** | Sex: M / F | DOB ........................ | Domicile ............................................................. |
| Spouse : | Date of birth ............................................. | Language .......... | Tribe ........................... |
| Are the spouse related ? Yes / No | | | |

| | | | |
|---|---|---|---|
| **Eighth child :** | Sex: M / F | DOB ........................ | Domicile ............................................................. |
| Spouse : | Date of birth ............................................. | Language .......... | Tribe ........................... |
| Are the spouse related ? Yes / No | | | |

| | | | |
|---|---|---|---|
| **Ninth child :** | Sex: M / F | DOB ........................ | Domicile ............................................................. |
| Spouse : | Date of birth ............................................. | Language .......... | Tribe ........................... |
| Are the spouse related ? Yes / No | | | |

**.../...**

1. Verdu, P. *et al.* Patterns of Admixture and Population Structure in Native Populations of Northwest North America. *PLoS Genet.* **10,** e1004530 (1-17) (2014).
2. Michael Boehnke, N. J. C. Accute Inference of Relationships in Sib-Pair Linkage Studies. *Am J Hum Genet* **61,** 423–429 (1997).
3. Epstein, M. P., Duren, W. L. & Boehnke, M. Improved inference of relationship for pairs of individuals. *Am. J. Hum. Genet.* **67,** 1219–31 (2000).
4. Damgaard, P. de B. *et al.* 137 ancient human genomes from across the Eurasian steppes. *Nature* 1 (2018). doi:10.1038/s41586-018-0094-2
5. Chang, C. C. *et al.* Second-generation PLINK: rising to the challenge of larger and richer datasets. *Gigascience* **4,** 7 (2015).
6. Szpiech, Z. A. asd computer program. (2011).
7. Excoffier, L. & Lischer, H. E. L. Arlequin suite ver 3.5: a new series of programs to perform population genetics analyses under Linux and Windows. *Mol. Ecol. Resour.* **10,** 564–567 (2010).
8. R Core Team. R: A Language and Environment for Statistical Computing. (2014).
9. Purcell, S. *et al.* PLINK: A Tool Set for Whole-Genome Association and Population-Based Linkage Analyses. *Am. J. Hum. Genet.* **81,** 559–575 (2007).
10. Gazal, S., Génin, E. & Leutenegger, A.-L. Relationship inference from the genetic data on parents or offspring: A comparative study. *Theor. Popul. Biol.* (2015). doi:10.1016/j.tpb.2015.09.002
11. Gazal, S., Sahbatou, M., Babron, M.-C., Génin, E. & Leutenegger, A.-L. FSuite: exploiting inbreeding in dense SNP chip and exome data. *Bioinformatics* **30,** 1940–1941 (2014).
12. Pemberton, T. J., Wang, C., Li, J. Z. & Rosenberg, N. a. Inference of unexpected genetic

relatedness among individuals in HapMap phase III. *Am. J. Hum. Genet.* **87,** 457–464 (2010).

13. Pemberton, T. J. *et al.* Genomic patterns of homozygosity in worldwide human populations. *Am. J. Hum. Genet.* **91,** 275–292 (2012).

14. Joshi, P. K. *et al.* Directional dominance on stature and cognition in diverse human populations. *Nature* **523,** 459–62 (2015).

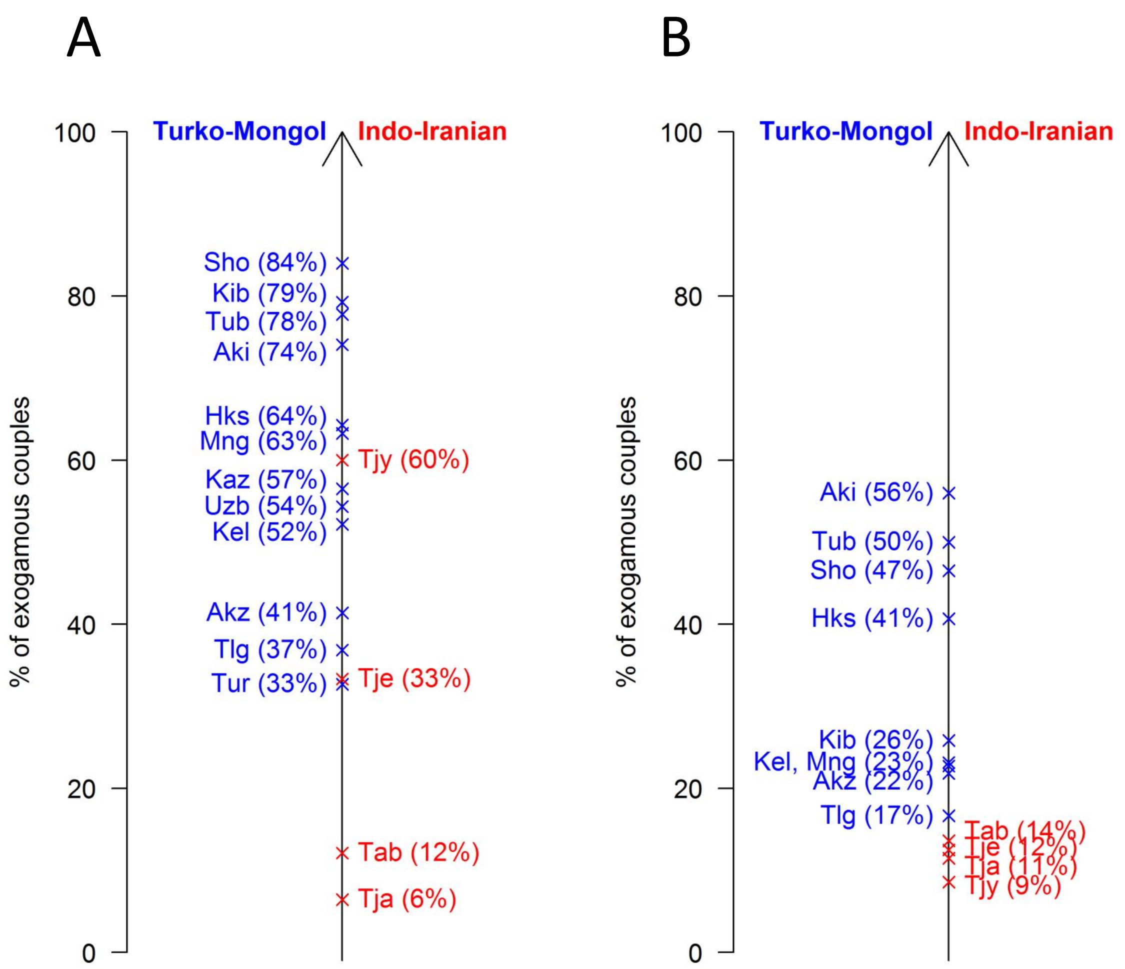


**Figure S1 – Population exogamy rate.**
Percentage of exogamous couples with a distance between the mates' birth places >4 km for the sampled couples (A), or their parents (B).

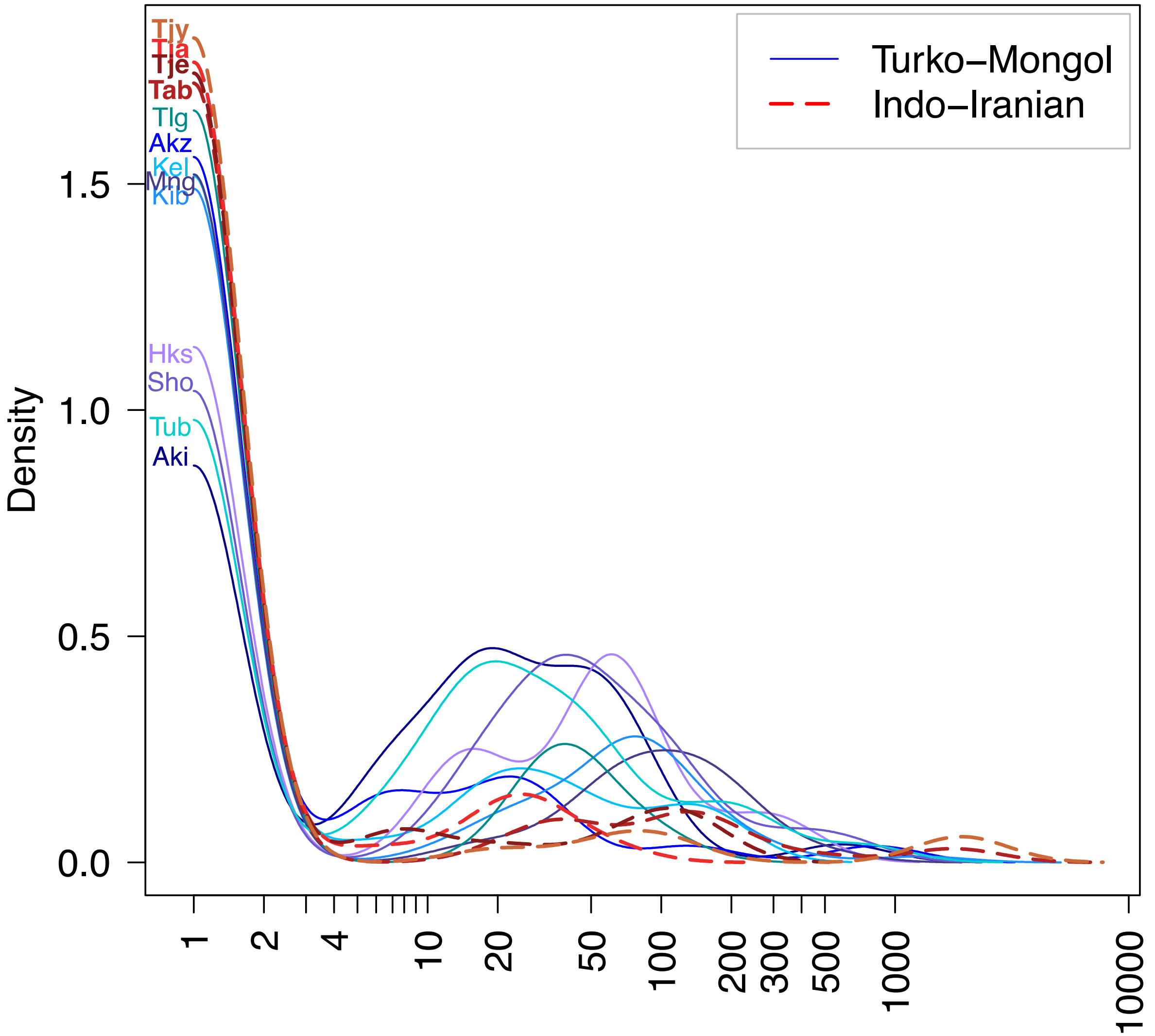


**Figure S2 – Geographical distances between parents' birth places.**
The distances are plotted in log scale (km).

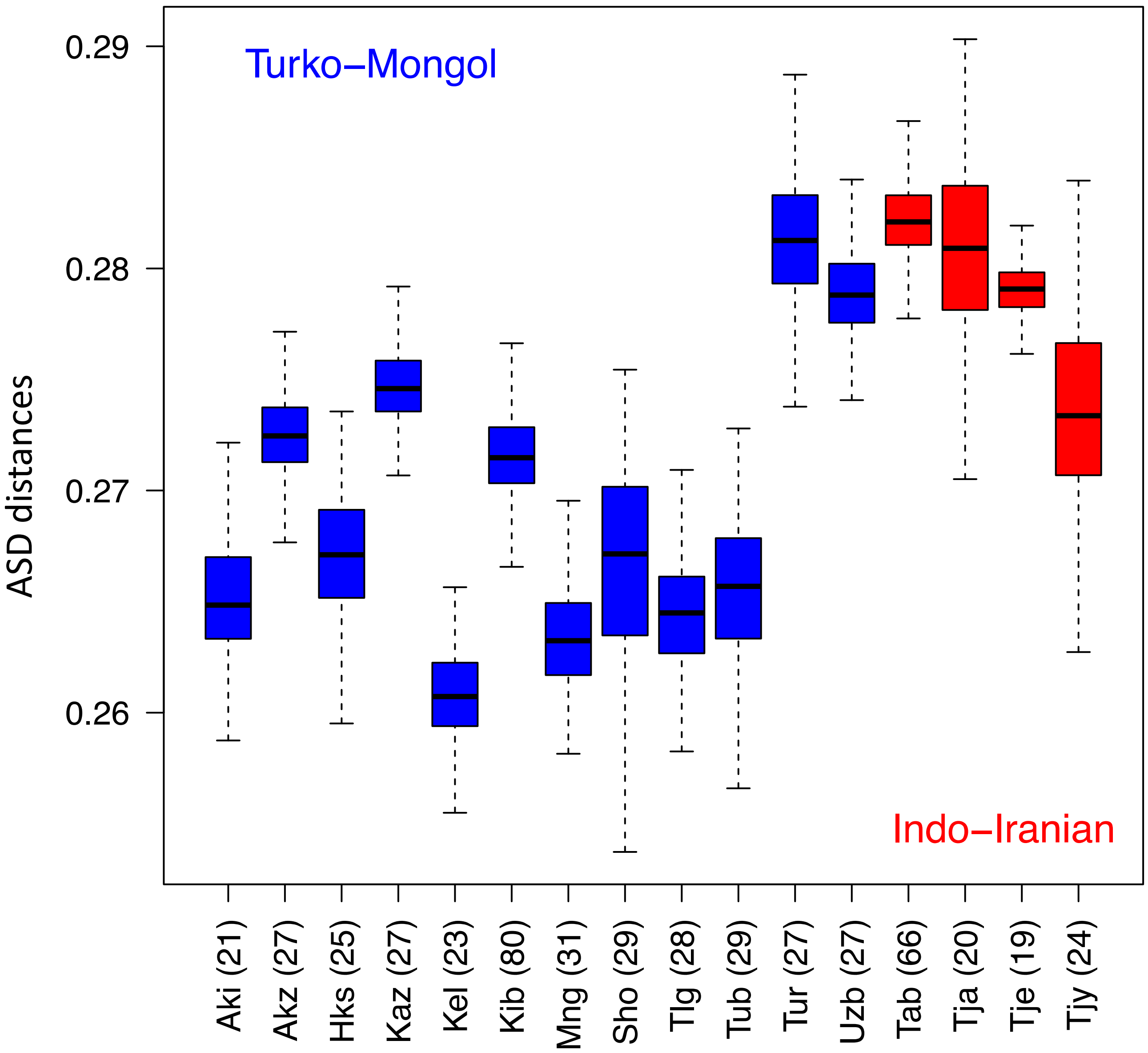


**Figure S3 – ASD distances computed for pairs of individuals within population.**
The number of samples by population is indicated after their name.

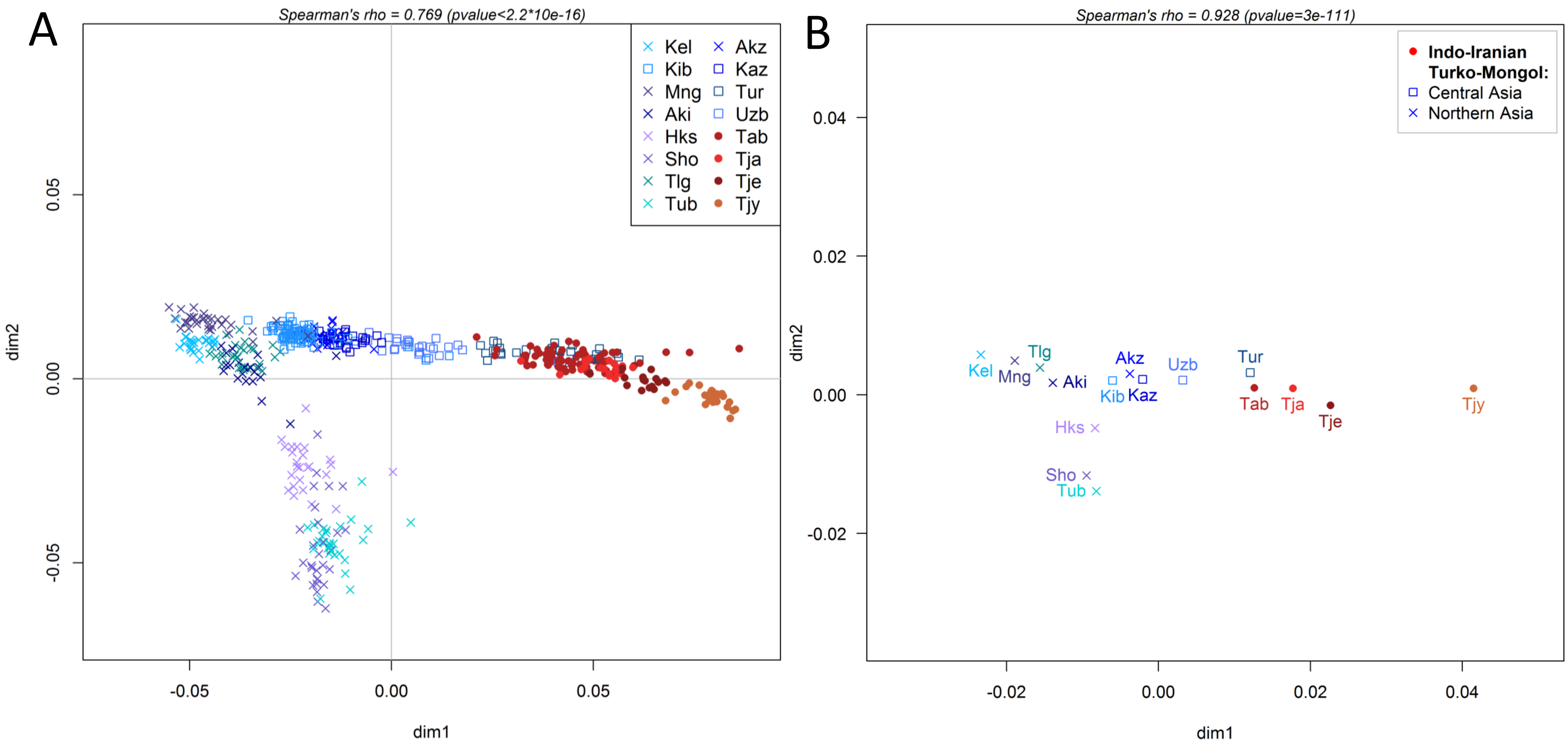


**Figure S4 – Multidimensional Scaling (MDS) plot over individuals pairwise ASD distances (A), and populations pairwise $F_{ST}$ distances (B).** Only the two first dimensions are represented. Each Spearman's coefficient of correlation (*rho*) was calculated between the MDS matrix and the pairwise genetic distances matrix.

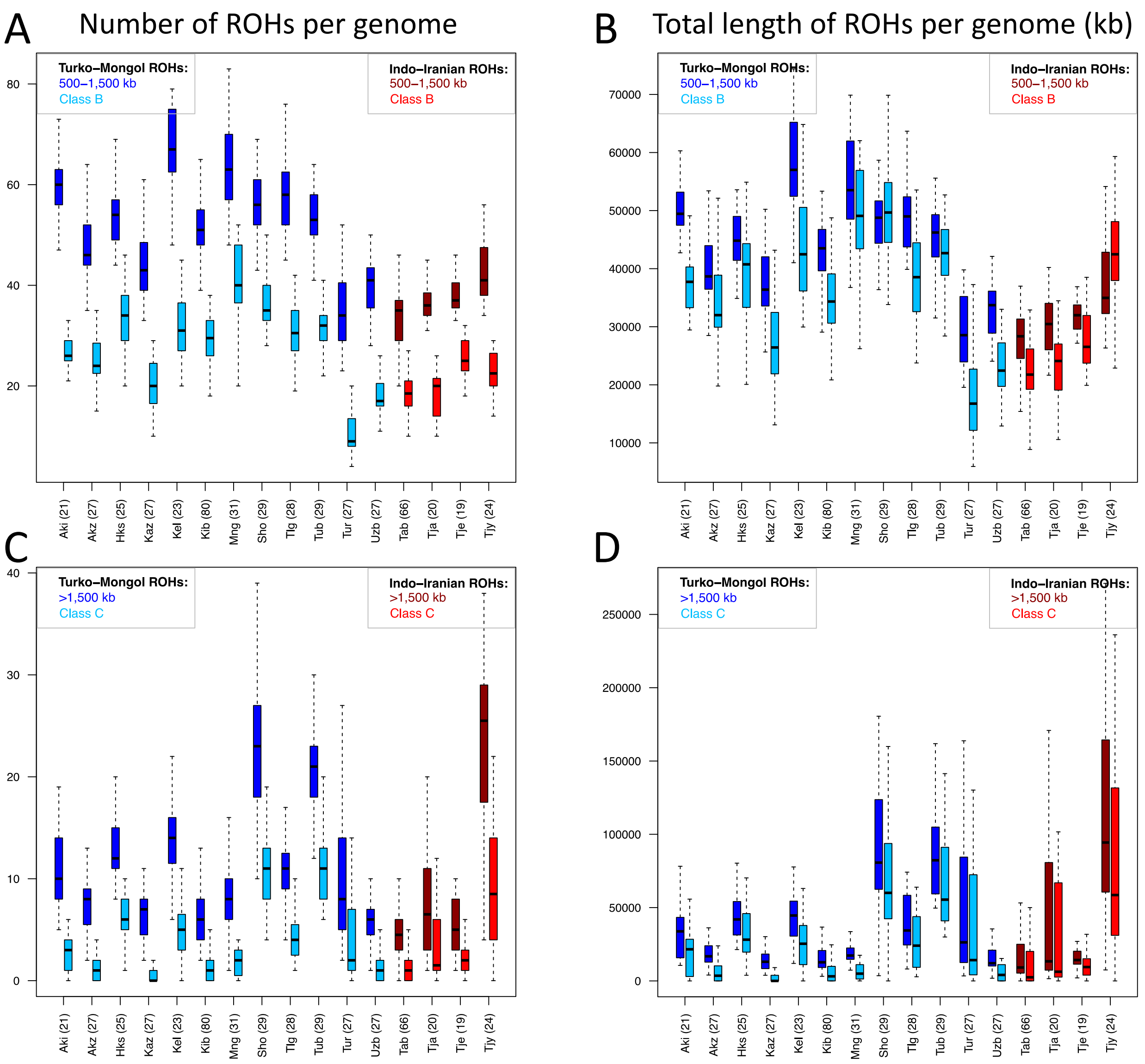


**Figure S5 – Populations pattern of intermediate sized ROHs (length between 500 and 1500 kb, or class B) (top) and long sized ROHs (larger than 1500 kb or class C) (bottom).**
Information is summarized by the number of segments per individual genome (A and C), or their total length by genome (B and D, in kb), and represented for each population. The number of genomes by population is indicated after their name.

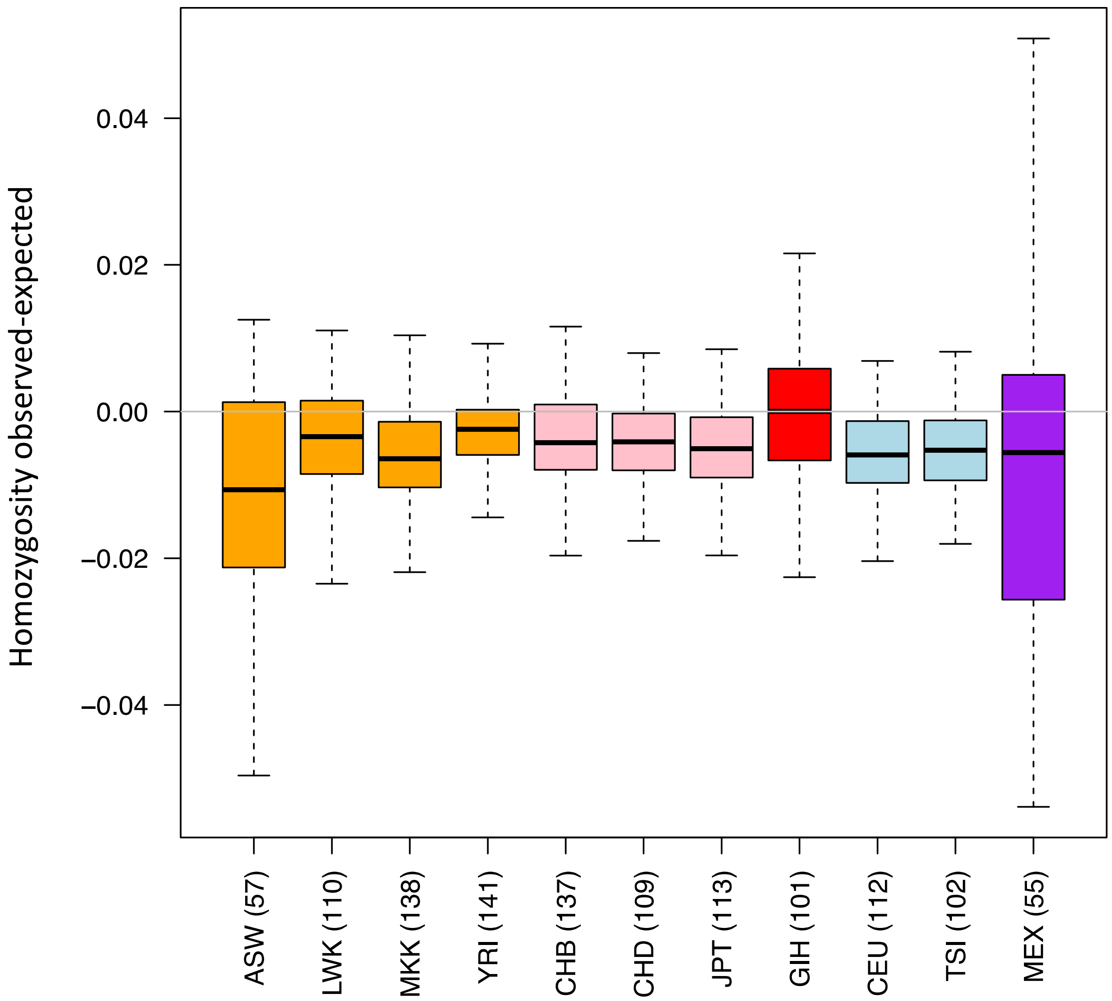


**Figure S6 – Inbreeding avoidance within HapMap3 populations**

Differences between the observed homozygosity within individual genomes and the population baseline, expected under panmixia. Number of samples by population is indicated after their name.

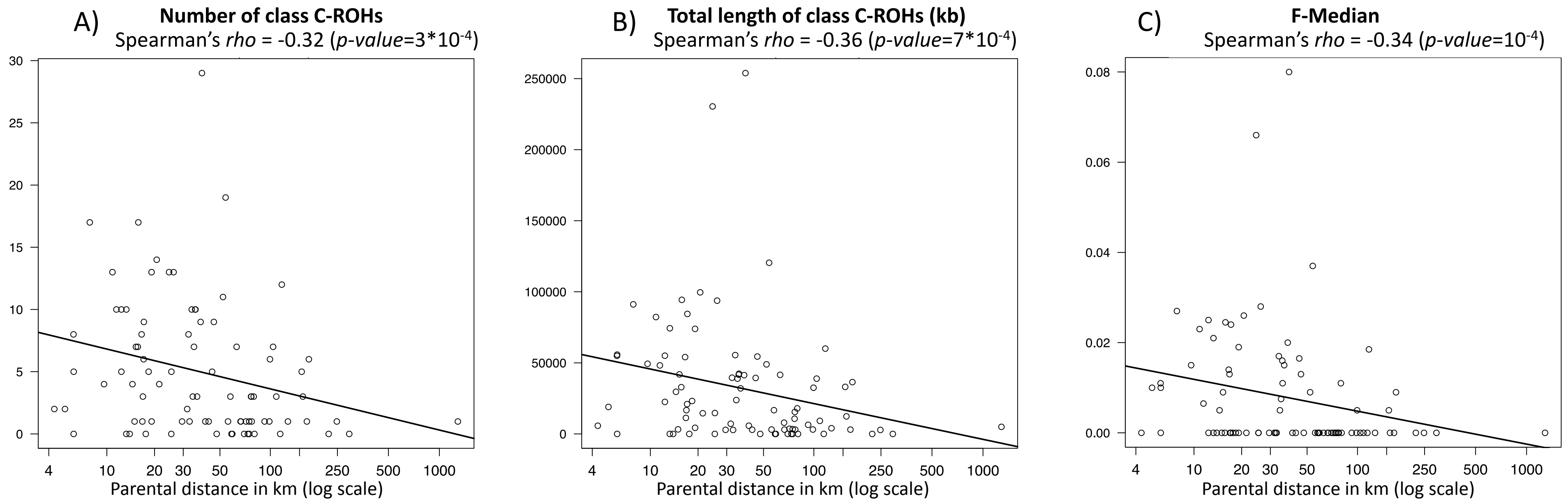


**Figure S7 – Inbreeding and parental couple distance for the offspring of exogamous Turko-Mongol couples.**
Three estimators of inbreeding are plotted against the distances between parents' birth places of exogamous couples. These two variables were correlated based on Spearman's correlation test (illustrated as a regression line on the plot).

**Table S1 A -** Genetic diversity, inbreeding and exogamy pattern within populations.

| Population: | | Aki | Akz | Hks | Kaz | Kel | Kib | Mng | Sho | Tlg | Tub | Tur | Uzb | Tab | Tja | Tje | Tjy |
|---|---|---|---|---|---|---|---|---|---|---|---|---|---|---|---|---|---|
| **Genetic dataset sample size (N)** | Females | 9 | 11 | 15 | 0 | 11 | 40 | 15 | 12 | 13 | 11 | 0 | 0 | 35 | 0 | 0 | 0 |
| | Males | 12 | 16 | 10 | 27 | 12 | 40 | 16 | 17 | 15 | 18 | 27 | 27 | 31 | 20 | 19 | 24 |
| | Couples | 4 | 4 | 2 | 0 | 8 | 38 | 14 | 5 | 12 | 4 | 0 | 0 | 24 | 0 | 0 | 0 |
| **ASD between individuals** | Mean distance | 0.228 | 0.236 | 0.230 | 0.238 | 0.224 | 0.235 | 0.227 | 0.229 | 0.223 | 0.228 | 0.247 | 0.243 | 0.249 | 0.247 | 0.246 | 0.240 |
| | *s.d.* | *0.005* | *0.003* | *0.003* | *0.002* | *0.004* | *0.002* | *0.003* | *0.005* | *0.004* | *0.005* | *0.004* | *0.002* | *0.002* | *0.005* | *0.005* | *0.007* |
| **Haplotypic heterozygosity** | Mean | 0.673 | 0.691 | 0.681 | 0.699 | 0.663 | 0.692 | 0.670 | 0.676 | 0.673 | 0.678 | 0.712 | 0.710 | 0.721 | 0.709 | 0.709 | 0.691 |
| | *s.d.* | *0.017* | *0.013* | *0.018* | *0.015* | *0.019* | *0.015* | *0.016* | *0.020* | *0.018* | *0.019* | *0.013* | *0.013* | *0.014* | *0.014* | *0.011* | *0.017* |
| **ROH limits** | Limit A-B (kb) | 931 | 863 | 791 | 946 | 928 | 808 | 773 | 859 | 847 | 870 | 1107 | 889 | 813 | 858 | 709 | 1117 |
| | Limit B-C (kb) | 2748 | 2768 | 2239 | 2896 | 2569 | 2596 | 2410 | 2641 | 2461 | 2546 | 3243 | 2604 | 2400 | 2490 | 2119 | 3642 |
| **Individual F-Median** | Median | 0.000 | 0.000 | 0.000 | 0.000 | 0.000 | 0.000 | 0.000 | 0.019 | 0.000 | 0.015 | 0.000 | 0.000 | 0.000 | 0.000 | 0.000 | 0.022 |
| | *s.d.* | *0.0066* | *0.0040* | *0.0089* | *0.0129* | *0.0067* | *0.0080* | *0.0030* | *0.0178* | *0.0073* | *0.0134* | *0.0232* | *0.0137* | *0.0122* | *0.0171* | *0.0014* | *0.0263* |
| | Positive F-Median (%) | 33% | 22% | 48% | 15% | 43% | 14% | 10% | 83% | 46% | 90% | 48% | 19% | 29% | 35% | 11% | 79% |
| **Type of parental relatedness** | Outbred (%) | 76 | 85 | 60 | 85 | 61 | 89 | 90 | 21 | 57 | 17 | 52 | 81 | 74 | 65 | 95 | 21 |
| | 2C-type (%) | 24 | 15 | 40 | 7 | 39 | 10 | 10 | 69 | 43 | 76 | 30 | 15 | 20 | 30 | 5 | 54 |
| | 1C-type (%) | 0 | 0 | 0 | 7 | 0 | 1 | 0 | 10 | 0 | 7 | 19 | 4 | 6 | 5 | 0 | 21 |
| | 2x1C-type (%) | 0 | 0 | 0 | 0 | 0 | 0 | 0 | 0 | 0 | 0 | 0 | 0 | 0 | 0 | 0 | 4 |
| **Individuals with a deficit in homozygosity** | | 100% (21/21) | 100% (27/27) | 96% (24/25) | 89% (24/27) | 91% (21/23) | 76% (61/80) | 90% (28/31) | 79% (23/29) | 93% (26/28) | 90% (26/29) | 70% (19/27) | 89% (24/27) | 68% (45/66) | 85% (17/20) | 100% (19/19) | 75% (18/24) |
| **Distances between partners' birth places** | Couples in the ethnological dataset (N) | 27 | 29 | 28 | 46 | 23 | 82 | 49 | 25 | 19 | 27 | 52 | 46 | 66 | 31 | 27 | 35 |
| | Median (km) | 15 | 0 | 22 | 40 | 13 | 48 | 58 | 46 | 0 | 23 | 0 | 12 | 0 | 0 | 0 | 18 |
| | 0-95% (km) | 0-67 | 0-26 | 0-123 | 0-607 | 0-149 | 0-261 | 0-180 | 0-252 | 0-74 | 0-576 | 0-163 | 0-264 | 0-43 | 0-3 | 0-137 | 0-111 |
| | Exogamous couples (%) | 74 | 41 | 64 | 57 | 52 | 79 | 63 | 84 | 37 | 78 | 33 | 54 | 12 | 6 | 33 | 60 |

**Table S1 B –** Geographical and ethnological information about the populations under study.

| Population: | Aki | Akz | Hks | Kaz | Kel | Kib | Mng | Sho | Tlg | Tub | Tur | Uzb | Tab | Tja | Tje | Tjy |
|---|---|---|---|---|---|---|---|---|---|---|---|---|---|---|---|---|
| **Cultural group** | Turko-Mongol | Turko-Mongol | Turko-Mongol | Turko-Mongol | Turko-Mongol | Turko-Mongol | Turko-Mongol | Turko-Mongol | Turko-Mongol | Turko-Mongol | Turko-Mongol | Turko-Mongol | Indo-Iranian | Indo-Iranian | Indo-Iranian | Indo-Iranian |
| **Ethnic Group** | Altai-Kizhi | Kazakh | Khakas | Kazakh | Kyrgyz | Kyrgyz | Mongol | Shor | Telengit | Tubalar | Turkmen | Uzbek | Tajik | Tajik | Tajik | Tajik-Yagnob |
| **Location** | Kulady, Russia | Kosh-Agach, Russia | Kazanovka, Russia | Qongirat area, UZB | Erzin & Chagonar area, Russia | Bishkek, KYR. | Khovd, Mongolia | Mezhdur-echensk, Russia | Kokorja, Russia | Kebezen, Russia | Tortkul area, UZB | Qongirat area, UZB | Bukhara, UZB. | Agalyk, UZB | Nimich, TAJ | Dughoba, TAJ |
| **Altitude (m)** | 1150 | 1784 | 473 | 59 | 1028 | 755 | 1916 | 261 | 1857 | 420 | 101 | 59 | 229 | 825 | 2064 | 825 |
| **Ecosystem** | Steppe | Steppe | Steppe | Desert | Steppe | Plain | Steppe | Forest | Steppe | Forest | Desert | Desert | Plain | Mountain | Mountain | Mountain |
| **Lifestyle** | Rural | Rural | Rural | Rural | Rural | Urban | Urban | Rural | Rural | Rural | Rural | Rural | Urban | Rural | Rural | Rural |
| | Traditionally; Turko-Mongol are semi-nomadic herders. | | | | | | | | | | | | Traditionally; Indo-Iranians are sedentary agriculturists | | | |

*UZB: Uzbekistan, KYR: Kyrgyzstan, TAJ: Tajikistan.*

**Table S2 -** Fst pairwise differences

| | Aki | Akz | Bou | Hks | Kaz | Kel | Kib | Mng | Sho | Tab | Tja | Tje | Tjy | Tlg | Tub | Tur | Uzb |
|---|---|---|---|---|---|---|---|---|---|---|---|---|---|---|---|---|---|
| **Aki** | 0 | 0.00784 | 0.0141 | 0.01318 | 0.00759 | 0.00832 | 0.00581 | 0.00694 | 0.01977 | 0.0297 | 0.03801 | 0.04463 | 0.06432 | 0.00674 | 0.01975 | 0.02978 | 0.01233 |
| **Akz** | 0.00784 | 0 | 0.01418 | 0.01083 | 0.00194 | 0.01096 | 0.00194 | 0.00609 | 0.01739 | 0.01716 | 0.02459 | 0.02993 | 0.04887 | 0.00876 | 0.01941 | 0.01772 | 0.00428 |
| **Bou** | 0.0141 | 0.01418 | 0 | 0.02251 | 0.01487 | 0.01218 | 0.01095 | 0.00784 | 0.03033 | 0.04484 | 0.05491 | 0.06336 | 0.0837 | 0.01456 | 0.03369 | 0.04465 | 0.02233 |
| **Hks** | 0.01318 | 0.01083 | 0.02251 | 0 | 0.01016 | 0.01591 | 0.00986 | 0.01458 | 0.00971 | 0.02488 | 0.03199 | 0.03702 | 0.05602 | 0.01436 | 0.01964 | 0.02582 | 0.01225 |
| **Kaz** | 0.00759 | 0.00194 | 0.01487 | 0.01016 | 0 | 0.01094 | 0.00151 | 0.00639 | 0.01625 | 0.01426 | 0.02113 | 0.02637 | 0.04483 | 0.0087 | 0.0179 | 0.01499 | 0.00221 |
| **Kel** | 0.00832 | 0.01096 | 0.01218 | 0.01591 | 0.01094 | 0 | 0.00767 | 0.00559 | 0.02376 | 0.03876 | 0.04828 | 0.0559 | 0.07631 | 0.00803 | 0.0272 | 0.03847 | 0.0179 |
| **Kib** | 0.00581 | 0.00194 | 0.01095 | 0.00986 | 0.00151 | 0.00767 | 0 | 0.00327 | 0.01628 | 0.01979 | 0.02728 | 0.03317 | 0.05199 | 0.00648 | 0.0184 | 0.02008 | 0.00487 |
| **Mng** | 0.00694 | 0.00609 | 0.00784 | 0.01458 | 0.00639 | 0.00559 | 0.00327 | 0 | 0.0221 | 0.03462 | 0.04399 | 0.05191 | 0.07216 | 0.00739 | 0.02504 | 0.0343 | 0.0134 |
| **Sho** | 0.01977 | 0.01739 | 0.03033 | 0.00971 | 0.01625 | 0.02376 | 0.01628 | 0.0221 | 0 | 0.02865 | 0.03565 | 0.04011 | 0.05865 | 0.02127 | 0.02091 | 0.02996 | 0.01759 |
| **Tab** | 0.0297 | 0.01716 | 0.04484 | 0.02488 | 0.01426 | 0.03876 | 0.01979 | 0.03462 | 0.02865 | 0 | 0.00429 | 0.00472 | 0.01918 | 0.03131 | 0.02869 | 0.00383 | 0.00651 |
| **Tja** | 0.03801 | 0.02459 | 0.05491 | 0.03199 | 0.02113 | 0.04828 | 0.02728 | 0.04399 | 0.03565 | 0.00429 | 0 | 0.00676 | 0.02183 | 0.04004 | 0.03537 | 0.00817 | 0.01245 |
| **Tje** | 0.04463 | 0.02993 | 0.06336 | 0.03702 | 0.02637 | 0.0559 | 0.03317 | 0.05191 | 0.04011 | 0.00472 | 0.00676 | 0 | 0.01975 | 0.04686 | 0.03994 | 0.00932 | 0.01615 |
| **Tjy** | 0.06432 | 0.04887 | 0.0837 | 0.05602 | 0.04483 | 0.07631 | 0.05199 | 0.07216 | 0.05865 | 0.01918 | 0.02183 | 0.01975 | 0 | 0.06654 | 0.05766 | 0.02475 | 0.03336 |
| **Tlg** | 0.00674 | 0.00876 | 0.01456 | 0.01436 | 0.0087 | 0.00803 | 0.00648 | 0.00739 | 0.02127 | 0.03131 | 0.04004 | 0.04686 | 0.06654 | 0 | 0.02297 | 0.03143 | 0.01354 |
| **Tub** | 0.01975 | 0.01941 | 0.03369 | 0.01964 | 0.0179 | 0.0272 | 0.0184 | 0.02504 | 0.02091 | 0.02869 | 0.03537 | 0.03994 | 0.05766 | 0.02297 | 0 | 0.02998 | 0.01869 |
| **Tur** | 0.02978 | 0.01772 | 0.04465 | 0.02582 | 0.01499 | 0.03847 | 0.02008 | 0.0343 | 0.02996 | 0.00383 | 0.00817 | 0.00932 | 0.02475 | 0.03143 | 0.02998 | 0 | 0.00801 |
| **Uzb** | 0.01233 | 0.00428 | 0.02233 | 0.01225 | 0.00221 | 0.0179 | 0.00487 | 0.0134 | 0.01759 | 0.00651 | 0.01245 | 0.01615 | 0.03336 | 0.01354 | 0.01869 | 0.00801 | 0 |

All pairwise distances were significant with *p-values* = 0.00000 (+-0.0000) for 1023 permutations.

**Table S3 -** Description of the genotyped dataset

| SampleID | Sex | Array |
|---|---|---|
| AKI001 | 2 | Omni1Quad |
| AKI002 | 1 | Omni1Quad |
| AKI009 | 2 | Omni1Quad |
| AKI010 | 1 | Omni1Quad |
| AKI011 | 1 | Omni1Quad |
| AKI014 | 2 | Omni1Quad |
| AKI016 | 2 | Omni1Quad |
| AKI022 | 1 | Omni1Quad |
| AKI024 | 2 | Omni1Quad |
| AKI026 | 1 | Omni1Quad |
| AKI027 | 1 | Omni1Quad |
| AKI028 | 2 | Omni1Quad |
| AKI031 | 1 | Omni1Quad |
| AKI032 | 2 | Omni1Quad |
| AKI036 | 1 | Omni1Quad |
| AKI037 | 2 | Omni1Quad |
| AKI038 | 1 | Omni1Quad |
| AKI039 | 1 | Omni1Quad |
| AKI042 | 2 | Omni1Quad |
| AKI043 | 1 | Omni1Quad |
| AKI045 | 1 | Omni1Quad |
| AKZ002 | 1 | Omni1Quad |
| AKZ003 | 1 | Omni1Quad |
| AKZ004 | 2 | Omni1Quad |
| AKZ005 | 2 | Omni1Quad |
| AKZ006 | 1 | Omni1Quad |
| AKZ009 | 1 | Omni1Quad |
| AKZ010 | 2 | Omni1Quad |
| AKZ012 | 2 | Omni1Quad |
| AKZ013 | 2 | Omni1Quad |
| AKZ014 | 1 | Omni1Quad |
| AKZ015 | 2 | Omni1Quad |
| AKZ016 | 1 | Omni1Quad |
| AKZ017 | 2 | Omni1Quad |
| AKZ018 | 1 | Omni1Quad |
| AKZ019 | 2 | Omni1Quad |
| AKZ020 | 1 | Omni1Quad |
| AKZ021 | 1 | Omni1Quad |
| AKZ022 | 1 | Omni1Quad |
| AKZ026 | 1 | Omni1Quad |
| AKZ028 | 2 | Omni1Quad |
| AKZ029 | 1 | Omni1Quad |
| AKZ030 | 2 | Omni1Quad |

| | | |
|---|---|---|
| AKZ035 | 1 | Omni1Quad |
| AKZ038 | 1 | Omni1Quad |
| AKZ040 | 2 | Omni1Quad |
| AKZ043 | 1 | Omni1Quad |
| AKZ044 | 1 | Omni1Quad |
| HKS003 | 2 | Omni1Quad |
| HKS004 | 2 | Omni1Quad |
| HKS007 | 2 | Omni1Quad |
| HKS008 | 2 | Omni1Quad |
| HKS009 | 1 | Omni1Quad |
| HKS010 | 2 | Omni1Quad |
| HKS011 | 2 | Omni1Quad |
| HKS013 | 2 | Omni1Quad |
| HKS014 | 2 | Omni1Quad |
| HKS016 | 2 | Omni1Quad |
| HKS017 | 2 | Omni1Quad |
| HKS019 | 1 | Omni1Quad |
| HKS020 | 2 | Omni1Quad |
| HKS021 | 2 | Omni1Quad |
| HKS023 | 2 | Omni1Quad |
| HKS024 | 2 | Omni1Quad |
| HKS026 | 1 | Omni1Quad |
| HKS027 | 2 | Omni1Quad |
| HKS028 | 1 | Omni1Quad |
| HKS034 | 1 | Omni1Quad |
| HKS035 | 1 | Omni1Quad |
| HKS038 | 1 | Omni1Quad |
| HKS039 | 1 | Omni1Quad |
| HKS042 | 1 | Omni1Quad |
| HKS043 | 1 | Omni1Quad |
| KAZ001 | 1 | Omni1Quad |
| KAZ002 | 1 | Omni1Quad |
| KAZ003 | 1 | Omni1Quad |
| KAZ004 | 1 | Omni1Quad |
| KAZ005 | 1 | Omni1Quad |
| KAZ006 | 1 | Omni1Quad |
| KAZ007 | 1 | Omni1Quad |
| KAZ008 | 1 | Omni1Quad |
| KAZ013 | 1 | Omni1Quad |
| KAZ015 | 1 | Omni1Quad |
| KAZ016 | 1 | Omni1Quad |
| KAZ017 | 1 | Omni1Quad |
| KAZ018 | 1 | Omni1Quad |
| KAZ019 | 1 | Omni1Quad |
| KAZ021 | 1 | Omni1Quad |
| KAZ022 | 1 | Omni1Quad |

| | | |
|---|---|---|
| KAZ025 | 1 | Omni1Quad |
| KAZ026 | 1 | Omni1Quad |
| KAZ028 | 1 | Omni1Quad |
| KAZ030 | 1 | Omni1Quad |
| KAZ032 | 1 | Omni1Quad |
| KAZ033 | 1 | Omni1Quad |
| KAZ036 | 1 | Omni1Quad |
| KAZ038 | 1 | Omni1Quad |
| KAZ039 | 1 | Omni1Quad |
| KAZ040 | 1 | Omni1Quad |
| KAZ042 | 1 | Omni1Quad |
| KEK001 | 2 | Omni2.5 |
| KEK002 | 1 | Omni2.5 |
| KEK003 | 2 | Omni2.5 |
| KEK006 | 1 | Omni2.5 |
| KEK007 | 1 | Omni2.5 |
| KEK008 | 2 | Omni2.5 |
| KEK009 | 2 | Omni2.5 |
| KEK010 | 1 | Omni2.5 |
| KEK014 | 1 | Omni2.5 |
| KEK015 | 2 | Omni2.5 |
| KEK018 | 1 | Omni2.5 |
| KEK019 | 2 | Omni2.5 |
| KEM001 | 2 | Omni2.5 |
| KEM002 | 1 | Omni2.5 |
| KEM007 | 1 | Omni2.5 |
| KEM008 | 2 | Omni2.5 |
| KEM009 | 2 | Omni2.5 |
| KEM010 | 1 | Omni2.5 |
| KEM013 | 2 | Omni2.5 |
| KEM016 | 1 | Omni2.5 |
| KEM017 | 1 | Omni2.5 |
| KEM019 | 2 | Omni2.5 |
| KEM020 | 1 | Omni2.5 |
| KIB001 | 2 | 660W-Quad |
| KIB003 | 2 | Omni1Quad |
| KIB004 | 1 | Omni1Quad |
| KIB005 | 1 | Omni1Quad |
| KIB006 | 2 | Omni1Quad |
| KIB007 | 2 | 660W-Quad |
| KIB008 | 1 | 660W-Quad |
| KIB010 | 1 | Omni1Quad |
| KIB011 | 2 | Omni1Quad |
| KIB012 | 2 | 660W-Quad |
| KIB013 | 1 | 660W-Quad |
| KIB015 | 2 | Omni1Quad |

| | | |
|---|---|---|
| KIB016 | 1 | 660W-Quad |
| KIB017 | 1 | 660W-Quad |
| KIB018 | 2 | 660W-Quad |
| KIB021 | 1 | 660W-Quad |
| KIB022 | 1 | 660W-Quad |
| KIB024 | 2 | 660W-Quad |
| KIB025 | 1 | Omni1Quad |
| KIB026 | 2 | Omni1Quad |
| KIB027 | 1 | 660W-Quad |
| KIB028 | 1 | 660W-Quad |
| KIB029 | 1 | 660W-Quad |
| KIB030 | 1 | 660W-Quad |
| KIB031 | 2 | 660W-Quad |
| KIB032 | 2 | 660W-Quad |
| KIB033 | 1 | 660W-Quad |
| KIB034 | 2 | Omni1Quad |
| KIB035 | 1 | 660W-Quad |
| KIB036 | 2 | 660W-Quad |
| KIB037 | 2 | Omni1Quad |
| KIB038 | 1 | 660W-Quad |
| KIB039 | 1 | Omni1Quad |
| KIB040 | 2 | 660W-Quad |
| KIB041 | 1 | 660W-Quad |
| KIB042 | 1 | 660W-Quad |
| KIB043 | 1 | Omni1Quad |
| KIB044 | 2 | 660W-Quad |
| KIB045 | 2 | Omni1Quad |
| KIB046 | 2 | Omni1Quad |
| KIB047 | 1 | Omni1Quad |
| KIB049 | 2 | 660W-Quad |
| KIB050 | 2 | Omni1Quad |
| KIB051 | 2 | Omni1Quad |
| KIB052 | 1 | Omni1Quad |
| KIB053 | 2 | Omni1Quad |
| KIB054 | 1 | 660W-Quad |
| KIB057 | 2 | Omni1Quad |
| KIB058 | 1 | Omni1Quad |
| KIB060 | 1 | Omni1Quad |
| KIB061 | 2 | Omni1Quad |
| KIB064 | 1 | Omni1Quad |
| KIB065 | 2 | Omni1Quad |
| KIB066 | 1 | Omni1Quad |
| KIB067 | 2 | Omni1Quad |
| KIB068 | 1 | Omni1Quad |
| KIB070 | 2 | 660W-Quad |
| KIB071 | 1 | 660W-Quad |

| | | |
|---|---|---|
| KIB072 | 2 | 660W-Quad |
| KIB074 | 1 | 660W-Quad |
| KIB077 | 2 | 660W-Quad |
| KIB083 | 2 | 660W-Quad |
| KIB084 | 1 | 660W-Quad |
| KIB092 | 2 | 660W-Quad |
| KIB093 | 1 | 660W-Quad |
| KIB103 | 1 | 660W-Quad |
| KIB104 | 2 | 660W-Quad |
| KIB105 | 1 | Omni1Quad |
| KIB106 | 2 | Omni1Quad |
| KIB107 | 1 | 660W-Quad |
| KIB109 | 2 | 660W-Quad |
| KIB110 | 1 | Omni1Quad |
| KIB126 | 1 | Omni1Quad |
| KIB127 | 2 | 660W-Quad |
| KIB128 | 1 | 660W-Quad |
| KIB136 | 2 | 660W-Quad |
| KIB138 | 2 | 660W-Quad |
| KIB149 | 2 | 660W-Quad |
| KIB152 | 1 | 660W-Quad |
| KIB153 | 2 | Omni1Quad |
| MNG014 | 2 | Omni2.5 |
| MNG015 | 1 | Omni2.5 |
| MNG048 | 1 | Omni2.5 |
| MNG054 | 2 | Omni2.5 |
| MNG055 | 1 | Omni2.5 |
| MNG057 | 2 | Omni2.5 |
| MNG058 | 1 | Omni2.5 |
| MNG059 | 2 | Omni2.5 |
| MNG060 | 2 | Omni2.5 |
| MNG061 | 1 | Omni2.5 |
| MNG062 | 2 | Omni2.5 |
| MNG063 | 1 | Omni2.5 |
| MNG066 | 2 | Omni2.5 |
| MNG067 | 1 | Omni2.5 |
| MNG070 | 1 | Omni2.5 |
| MNG071 | 2 | Omni2.5 |
| MNG072 | 1 | Omni2.5 |
| MNG073 | 2 | Omni2.5 |
| MNG074 | 1 | Omni2.5 |
| MNG075 | 2 | Omni2.5 |
| MNG076 | 1 | Omni2.5 |
| MNG077 | 2 | Omni2.5 |
| MNG078 | 1 | Omni2.5 |
| MNG079 | 1 | Omni2.5 |

| | | |
|---|---|---|
| MNG080 | 1 | Omni2.5 |
| MNG081 | 2 | Omni2.5 |
| MNG082 | 1 | Omni2.5 |
| MNG083 | 2 | Omni2.5 |
| MNG088 | 2 | Omni2.5 |
| MNG089 | 1 | Omni2.5 |
| MNG090 | 1 | Omni2.5 |
| SHO002 | 1 | Omni1Quad |
| SHO003 | 2 | Omni1Quad |
| SHO004 | 2 | Omni1Quad |
| SHO006 | 2 | Omni1Quad |
| SHO007 | 1 | Omni1Quad |
| SHO008 | 1 | Omni1Quad |
| SHO011 | 1 | Omni1Quad |
| SHO013 | 2 | Omni1Quad |
| SHO014 | 1 | Omni1Quad |
| SHO015 | 1 | Omni1Quad |
| SHO016 | 2 | Omni1Quad |
| SHO019 | 1 | Omni1Quad |
| SHO020 | 2 | Omni1Quad |
| SHO021 | 2 | Omni1Quad |
| SHO022 | 2 | Omni1Quad |
| SHO023 | 1 | Omni1Quad |
| SHO024 | 1 | Omni1Quad |
| SHO025 | 1 | Omni1Quad |
| SHO026 | 1 | Omni1Quad |
| SHO027 | 2 | Omni1Quad |
| SHO029 | 2 | Omni1Quad |
| SHO034 | 2 | Omni1Quad |
| SHO035 | 1 | Omni1Quad |
| SHO036 | 2 | Omni1Quad |
| SHO038 | 1 | Omni1Quad |
| SHO039 | 1 | Omni1Quad |
| SHO040 | 1 | Omni1Quad |
| SHO041 | 1 | Omni1Quad |
| SHO042 | 1 | Omni1Quad |
| TAB001 | 2 | 660W-Quad |
| TAB002 | 1 | 660W-Quad |
| TAB005 | 1 | Omni1Quad |
| TAB006 | 1 | 660W-Quad |
| TAB009 | 2 | 660W-Quad |
| TAB011 | 2 | 660W-Quad |
| TAB012 | 2 | 660W-Quad |
| TAB013 | 1 | Omni1Quad |
| TAB014 | 2 | 660W-Quad |
| TAB015 | 1 | 660W-Quad |

| | | |
|---|---|---|
| TAB017 | 2 | 660W-Quad |
| TAB018 | 1 | 660W-Quad |
| TAB019 | 2 | Omni1Quad |
| TAB020 | 1 | 660W-Quad |
| TAB021 | 2 | 660W-Quad |
| TAB022 | 1 | 660W-Quad |
| TAB023 | 2 | 660W-Quad |
| TAB024 | 1 | 660W-Quad |
| TAB028 | 1 | 660W-Quad |
| TAB029 | 2 | 660W-Quad |
| TAB031 | 2 | 660W-Quad |
| TAB036 | 2 | 660W-Quad |
| TAB041 | 1 | 660W-Quad |
| TAB042 | 2 | 660W-Quad |
| TAB043 | 1 | 660W-Quad |
| TAB045 | 1 | Omni1Quad |
| TAB046 | 2 | 660W-Quad |
| TAB051 | 2 | Omni1Quad |
| TAB052 | 1 | Omni1Quad |
| TAB055 | 1 | 660W-Quad |
| TAB056 | 2 | 660W-Quad |
| TAB057 | 2 | Omni1Quad |
| TAB058 | 1 | 660W-Quad |
| TAB059 | 1 | Omni1Quad |
| TAB060 | 2 | Omni1Quad |
| TAB061 | 1 | Omni1Quad |
| TAB062 | 2 | Omni1Quad |
| TAB063 | 1 | 660W-Quad |
| TAB064 | 2 | 660W-Quad |
| TAB066 | 2 | 660W-Quad |
| TAB074 | 2 | Omni1Quad |
| TAB075 | 1 | 660W-Quad |
| TAB076 | 2 | 660W-Quad |
| TAB077 | 2 | Omni1Quad |
| TAB078 | 1 | Omni1Quad |
| TAB079 | 1 | Omni1Quad |
| TAB080 | 2 | Omni1Quad |
| TAB081 | 1 | 660W-Quad |
| TAB082 | 1 | 660W-Quad |
| TAB087 | 1 | Omni1Quad |
| TAB088 | 2 | 660W-Quad |
| TAB092 | 2 | 660W-Quad |
| TAB103 | 2 | Omni1Quad |
| TAB104 | 1 | Omni1Quad |
| TAB106 | 1 | Omni1Quad |
| TAB107 | 2 | 660W-Quad |

| | | |
|---|---|---|
| TAB108 | 2 | 660W-Quad |
| TAB109 | 1 | 660W-Quad |
| TAB110 | 2 | 660W-Quad |
| TAB111 | 1 | Omni1Quad |
| TAB112 | 2 | Omni1Quad |
| TAB113 | 1 | Omni1Quad |
| TAB116 | 1 | Omni1Quad |
| TAB117 | 2 | Omni1Quad |
| TAB118 | 1 | Omni1Quad |
| TAB119 | 2 | Omni1Quad |
| TJA01 | 1 | OmniExpress |
| TJA02 | 1 | OmniExpress |
| TJA03 | 1 | OmniExpress |
| TJA04 | 1 | OmniExpress |
| TJA05 | 1 | OmniExpress |
| TJA06 | 1 | OmniExpress |
| TJA07 | 1 | OmniExpress |
| TJA08 | 1 | OmniExpress |
| TJA11 | 1 | OmniExpress |
| TJA12 | 1 | OmniExpress |
| TJA13 | 1 | OmniExpress |
| TJA14 | 1 | OmniExpress |
| TJA15 | 1 | OmniExpress |
| TJA16 | 1 | OmniExpress |
| TJA17 | 1 | OmniExpress |
| TJA18 | 1 | OmniExpress |
| TJA19 | 1 | OmniExpress |
| TJA20 | 1 | OmniExpress |
| TJA21 | 1 | OmniExpress |
| TJA_31 | 1 | Omni5Exome |
| TJE_1 | 1 | OmniExpress |
| TJE_10 | 1 | OmniExpress |
| TJE_11 | 1 | OmniExpress |
| TJE_12 | 1 | OmniExpress |
| TJE_13 | 1 | OmniExpress |
| TJE_14 | 1 | OmniExpress |
| TJE_15 | 1 | OmniExpress |
| TJE_16 | 1 | OmniExpress |
| TJE_17 | 1 | OmniExpress |
| TJE_18 | 1 | OmniExpress |
| TJE_19 | 1 | OmniExpress |
| TJE_21 | 1 | OmniExpress |
| TJE_22 | 1 | OmniExpress |
| TJE_4 | 1 | OmniExpress |
| TJE_5 | 1 | OmniExpress |
| TJE_6 | 1 | OmniExpress |

| | | |
|---|---|---|
| TJE_7 | 1 | OmniExpress |
| TJE_8 | 1 | OmniExpress |
| TJE_9 | 1 | OmniExpress |
| TJY02 | 1 | OmniExpress |
| TJY03 | 1 | OmniExpress |
| TJY05 | 1 | OmniExpress |
| TJY06 | 1 | OmniExpress |
| TJY07 | 1 | OmniExpress |
| TJY08 | 1 | OmniExpress |
| TJY10 | 1 | OmniExpress |
| TJY12 | 1 | OmniExpress |
| TJY15 | 1 | OmniExpress |
| TJY16 | 1 | OmniExpress |
| TJY17 | 1 | OmniExpress |
| TJY20 | 1 | OmniExpress |
| TJY22 | 1 | OmniExpress |
| TJY23 | 1 | OmniExpress |
| TJY24 | 1 | OmniExpress |
| TJY26 | 1 | Omni5Exome |
| TJY27 | 1 | Omni5Exome |
| TJY29 | 1 | Omni5Exome |
| TJY30 | 1 | Omni5Exome |
| TJY31 | 1 | Omni5Exome |
| TJY32 | 1 | Omni5Exome |
| TJY34 | 1 | Omni5Exome |
| TJY35 | 1 | Omni5Exome |
| TJY39 | 1 | Omni5Exome |
| TLG001 | 1 | Omni1Quad |
| TLG002 | 2 | Omni1Quad |
| TLG003 | 2 | Omni1Quad |
| TLG006 | 1 | Omni1Quad |
| TLG007 | 1 | Omni1Quad |
| TLG008 | 2 | Omni1Quad |
| TLG009 | 2 | Omni1Quad |
| TLG010 | 1 | Omni1Quad |
| TLG012 | 2 | Omni1Quad |
| TLG013 | 1 | Omni1Quad |
| TLG014 | 2 | Omni1Quad |
| TLG015 | 1 | Omni1Quad |
| TLG017 | 1 | Omni1Quad |
| TLG018 | 2 | Omni1Quad |
| TLG019 | 2 | Omni1Quad |
| TLG020 | 1 | Omni1Quad |
| TLG021 | 1 | Omni1Quad |
| TLG022 | 2 | Omni1Quad |
| TLG023 | 1 | Omni1Quad |

| | | |
|---|---|---|
| TLG024 | 2 | Omni1Quad |
| TLG025 | 2 | Omni1Quad |
| TLG026 | 1 | Omni1Quad |
| TLG029 | 2 | Omni1Quad |
| TLG030 | 1 | Omni1Quad |
| TLG031 | 2 | Omni1Quad |
| TLG032 | 1 | Omni1Quad |
| TLG034 | 1 | Omni1Quad |
| TLG036 | 1 | Omni1Quad |
| TUB001 | 1 | Omni1Quad |
| TUB002 | 1 | Omni1Quad |
| TUB003 | 2 | Omni1Quad |
| TUB004 | 1 | Omni1Quad |
| TUB006 | 2 | Omni1Quad |
| TUB007 | 2 | Omni1Quad |
| TUB008 | 2 | Omni1Quad |
| TUB009 | 2 | Omni1Quad |
| TUB010 | 1 | Omni1Quad |
| TUB013 | 1 | Omni1Quad |
| TUB014 | 2 | Omni1Quad |
| TUB015 | 2 | Omni1Quad |
| TUB018 | 2 | Omni1Quad |
| TUB024 | 1 | Omni1Quad |
| TUB025 | 2 | Omni1Quad |
| TUB026 | 1 | Omni1Quad |
| TUB027 | 2 | Omni1Quad |
| TUB028 | 1 | Omni1Quad |
| TUB030 | 1 | Omni1Quad |
| TUB031 | 1 | Omni1Quad |
| TUB032 | 1 | Omni1Quad |
| TUB033 | 1 | Omni1Quad |
| TUB034 | 1 | Omni1Quad |
| TUB035 | 2 | Omni1Quad |
| TUB036 | 1 | Omni1Quad |
| TUB037 | 1 | Omni1Quad |
| TUB038 | 1 | Omni1Quad |
| TUB039 | 1 | Omni1Quad |
| TUB040 | 1 | Omni1Quad |
| TUR001 | 1 | Omni1Quad |
| TUR007 | 1 | Omni1Quad |
| TUR008 | 1 | Omni1Quad |
| TUR011 | 1 | Omni1Quad |
| TUR012 | 1 | Omni1Quad |
| TUR013 | 1 | Omni1Quad |
| TUR016 | 1 | Omni1Quad |
| TUR018 | 1 | Omni1Quad |

| | | |
|---|---|---|
| TUR025 | 1 | Omni1Quad |
| TUR026 | 1 | Omni1Quad |
| TUR027 | 1 | Omni1Quad |
| TUR028 | 1 | Omni1Quad |
| TUR029 | 1 | Omni1Quad |
| TUR031 | 1 | Omni1Quad |
| TUR032 | 1 | Omni1Quad |
| TUR033 | 1 | Omni1Quad |
| TUR035 | 1 | Omni1Quad |
| TUR036 | 1 | Omni1Quad |
| TUR040 | 1 | Omni1Quad |
| TUR041 | 1 | Omni1Quad |
| TUR042 | 1 | Omni1Quad |
| TUR043 | 1 | Omni1Quad |
| TUR046 | 1 | Omni1Quad |
| TUR048 | 1 | Omni1Quad |
| TUR050 | 1 | Omni1Quad |
| TUR051 | 1 | Omni1Quad |
| TUR054 | 1 | Omni1Quad |
| UZB002 | 1 | Omni1Quad |
| UZB004 | 1 | Omni1Quad |
| UZB005 | 1 | Omni1Quad |
| UZB013 | 1 | Omni1Quad |
| UZB014 | 1 | Omni1Quad |
| UZB015 | 1 | Omni1Quad |
| UZB018 | 1 | Omni1Quad |
| UZB019 | 1 | Omni1Quad |
| UZB022 | 1 | Omni1Quad |
| UZB023 | 1 | Omni1Quad |
| UZB024 | 1 | Omni1Quad |
| UZB026 | 1 | Omni1Quad |
| UZB028 | 1 | Omni1Quad |
| UZB029 | 1 | Omni1Quad |
| UZB030 | 1 | Omni1Quad |
| UZB031 | 1 | Omni1Quad |
| UZB032 | 1 | Omni1Quad |
| UZB034 | 1 | Omni1Quad |
| UZB036 | 1 | Omni1Quad |
| UZB038 | 1 | Omni1Quad |
| UZB039 | 1 | Omni1Quad |
| UZB040 | 1 | Omni1Quad |
| UZB041 | 1 | Omni1Quad |
| UZB046 | 1 | Omni1Quad |
| UZB048 | 1 | Omni1Quad |
| UZB049 | 1 | Omni1Quad |
| UZB050 | 1 | Omni1Quad |
